# Espresso Coffee Mitigates the Aggregation and Condensation of Alzheimer′s Associated Tau Protein

**DOI:** 10.1021/acs.jafc.3c01072

**Published:** 2023-07-19

**Authors:** Roberto Tira, Giovanna Viola, Carlo Giorgio Barracchia, Francesca Parolini, Francesca Munari, Stefano Capaldi, Michael Assfalg, Mariapina D’Onofrio

**Affiliations:** Department of Biotechnology, University of Verona, Strada le Grazie 15, 34134 Verona, Italy

**Keywords:** tau protein, coffee, protein aggregation, NMR, bioactive molecules, liquid−liquid phase separation, Alzheimer′s disease

## Abstract

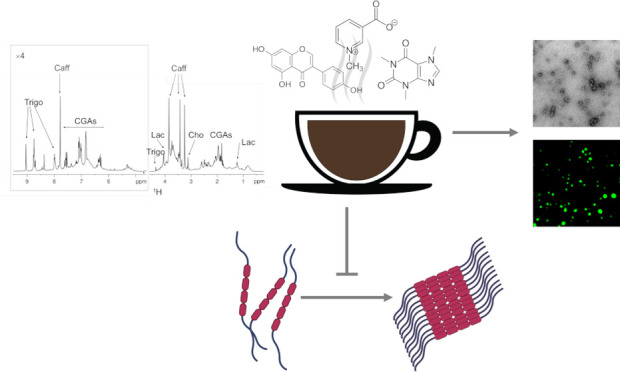

Espresso coffee is among the most consumed beverages in the world. Recent studies report a protective activity of the coffee beverage against neurodegenerative disorders such as Alzheimer′s disease. Alzheimer′s disease belongs to a group of disorders, called tauopathies, which are characterized by the intraneuronal accumulation of the microtubule-associated protein tau in fibrillar aggregates. In this work, we characterized by NMR the molecular composition of the espresso coffee extract and identified its main components. We then demonstrated with in vitro and in cell experiments that the whole coffee extract, caffeine, and genistein have biological properties in preventing aggregation, condensation, and seeding activity of the repeat region of tau. We also identified a set of coffee compounds capable of binding to preformed tau fibrils. These results add insights into the neuroprotective potential of espresso coffee and suggest candidate molecular scaffolds for designing therapies targeting monomeric or fibrillized forms of tau.

## Introduction

Espresso coffee is among the best known beverages worldwide, and drinking espresso has become a habit in many countries due to its pleasant taste. For many years, coffee consumption was associated with health risks; however, recent studies showed that when consumed in moderation, this soft drink could have beneficial effects on human health thanks to its biological properties.^1,2^ The analysis and review of observational studies present in the literature suggest that consuming coffee could be advantageous against a number of chronic diseases, including some cancers (liver, colorectal, endometrial, and prostate),^3^ metabolic diseases (type-2 diabetes and metabolic syndrome), and neurological disorders (Parkinson′s disease, Alzheimer′s disease, and depression).^1^ In particular, numerous studies report that moderate and, sometimes, even high coffee consumption exerts a neuroprotective action against two of the most common neurodegenerative diseases, i.e., Parkinson′s and Alzheimer′s.^4−6^ Many coffee compounds display beneficial properties in alleviating disease symptoms, for instance by reducing cognitive and memory impairment,^2^ as antioxidants,^7^ or by preventing amyloid formation and neurotoxicity.^8^ The coffee beverage consists of more than a thousand compounds; the beverage as a whole and its components show a bioactive role, and therefore, coffee is considered a potential functional food.

**Figure 1:**
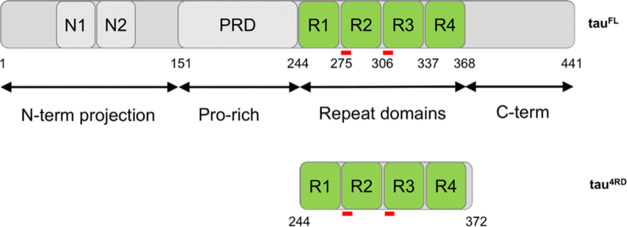
Domain organization of full-length tau, tau^FL^, and of the shorter construct comprising the four-repeat region, tau^4RD^. Red bars indicate the position of hexapeptide motifs known as aggregation nuclei.

Tauopathies is the term used to define a set of neurodegenerative disorders with symptoms of dementia and parkinsonism.^9^ Among the tauopathies identified so far, Alzheimer′s disease is the most common, with a worldwide prevalence of 50 million people, especially the elderly (age > 65 years). The primary feature of tauopathies is the abnormal accumulation of the microtubule-associated protein tau in the brain (neurons or glial cells or both). The mechanisms underlying the onset of these diseases are complex and currently unclear, but tau aggregation and spreading are thought to play a crucial role. The microtubule-associated protein tau (hereafter tau) binds to microtubules and regulates their assembly and axon outgrowth and integrity. Tau is expressed in six isoforms in the adult human brain, the most abundant being 441 amino acids long (Figure 1);^10^ it is an intrinsically disordered protein, highly soluble and with little tendency to aggregate. The repeat region is responsible for binding to microtubules and contains two hexapeptide motives, at the beginning of the second (R2) and third (R3) repeats, which drive tau aggregation (Figure 1). The dissociation of tau from microtubules is considered the leading cause of its pathological accumulation; however, the molecular mechanisms involved in the early events of pathogenesis are still not completely clear.^11^ Due to the increase in the elderly population, the number of patients with tauopathies expected in the future years is very high: this will represent a high socio-economic burden worldwide unless the means to prevent or treat these diseases are found. It is worth emphasizing that these diseases are currently incurable, as there are no effective disease-modifying treatments. In this complex scenario, nutraceuticals offer an attractive means for prevention strategies or for designing food-based therapeutics to interfere with the progression of the disease and mitigate its symptoms. Green and roasted coffee extracts and their main compounds have been previously investigated for their ability to target Aβ oligomers, involved in the progression of Alzheimer′s disease, hindering their fibrillization and neurotoxicity.^8^

In this work, we started with the NMR characterization of the molecular composition of the coffee extract to identify its main components. Next, we interrogated the biological properties of the whole coffee extract and of selected components, i.e., caffeine and genistein, and observed their ability to prevent aggregation, condensation, and the seeding activity of the tau protein. Moreover, we identified molecules among coffee compounds, able to bind to preformed fibrils of tau. Taken together, these results add insights into the neuroprotective potential of espresso coffee and suggest candidate molecular scaffolds for designing therapies targeting monomeric or fibrillized forms of the tau protein.

## Materials and Methods

### Chemicals

Trigonelline (analytical standard) was purchased from Sigma-Aldrich (St Louis, MO); caffeine (99% purity), genistein (99% purity), and theobromine (99% purity) were purchased from Alfa Aesar by Thermo Fisher Scientific (Kandel, Germany). Stock solutions of all compounds were prepared in mQ H_2_O at a concentration of 1 mg/mL and stored at −20 °C. All other reagents were purchased from Sigma-Aldrich (St Louis, MO) unless otherwise indicated.

### Espresso Extraction

The medium roast ground coffee used in this study is a blend of Arabica coffee from South America and Robusta coffee from Africa and Southwest Asia (commercial brand). The espresso coffee extract was obtained from 15 g of powder using a two-cups coffee machine (Gaggia espresso machine, Gaggia Milano, Italy) for a final volume of 80 mL of beverage. The extraction lasted for 30 s at 80 °C in mQ H_2_O. The final product was distributed in 15 mL Falcon tubes, freeze-dried, and stored at +4 °C.

### Recombinant Tau^4RD^ Expression and Purification

All tau^4RD^ variants were expressed in BL21(DE3) cells grown in LB medium, at 37 °C for 5 h with 0.5 mM IPTG. Protein purification was achieved by thermal treatment of the soluble bacterial extract (80 °C) followed by the SP-ion exchange chromatography step.^12−14^

### Thioflavin-T Aggregation Assay

Solutions of the 50 μM tau^4RD^ protein in 20 mM sodium phosphate buffer at pH 7.4, 50 mM NaCl, 1 mM DTT, 0.02% NaN_3_, and protease inhibitors with EDTA were incubated in the absence or presence of coffee extract or different compounds in 96-well dark plates at 37 °C for 40 h. Heparin and Thioflavin-T (ThT) were added to the sample solutions in a molar ratio of 1:1 with respect to the protein. Fluorescence measurements (λ*e*x.: 450 nm and *λe*m: 482 nm) were performed with a Tecan Infinite M200 Pro Microplate Reader (Tecan Group AG, Männedorf, Switzerland) with cycles of 30 s of orbital shaking at 140 rpm and 10 min of rest before the fluorescence reading throughout the incubation, as described in previous work.^15^ The fluorescence intensity and lag-phase duration of four replicates of each sample were analyzed with GraphPad Prism 8.2 software (GraphPad Software, San Diego, California, www.graphpad.com). Any pre-existing aggregate was removed by filtering the protein stock solutions through a 100 MWCO cut-off filter (Sartorius Stedim Biotech GmbH, Göttingen, Germany), before the aggregation reaction. Error bars of ThT curves correspond to standard deviations of four independent experiments.

### Sample Preparation for CD and TEM Analysis

Solutions of the 50 μM filtered tau^4RD^ protein in 20 mM sodium phosphate buffer at pH 7.4, 50 mM NaCl, 1 mM DTT, 0.02% NaN_3_, and protease inhibitors with EDTA were incubated in the absence or presence of coffee extract or different compounds in static conditions at 37 °C for 48/72 h using heparin at a 1:1 molar ratio as the aggregation initiator.

### CD Analysis

Circular dichroism (CD) spectra were collected using a Jasco J-1500 spectropolarimeter equipped with a Peltier-type cell holder for temperature control (Jasco, Easton, MD). Solutions containing tau^4RD^ aggregates were diluted in 20 mM sodium phosphate buffer, pH 7.4, to a final concentration of 6 μM. Far-UV spectra (190–260 nm) were recorded at 25 °C with a scan rate of 50 nm min^–1^, a bandwidth of 1 nm, and an integration time of 2 s, in 0.1 cm cuvettes. Three spectra accumulations were collected and averaged for each sample at different times (0 and 72 h). The spectrum of the buffer alone (with or without the compounds) was subtracted from the spectrum of the corresponding sample. Data were analyzed with Spectra Manager and graphs were generated with GraphPad Prism 8.2 software.

### TEM Analysis

For transmission electron microscopy (TEM) measurements, 10 μL of tau^4RD^ aggregates (obtained after 48 h incubation with or without coffee extract or different compounds) were washed and diluted in mQ H_2_O to a final concentration of 5 μM (monomer concentration). 30 μL of diluted aggregates were adsorbed onto a film grid (400 mesh) and stained for 2 min with 2% uranyl acetate. A Tecnai G2 (FEI) transmission electron microscope instrument operating at 100 kV was employed to analyze the samples. Images were acquired with a Veleta digital camera (Olympus Soft Imaging System, Münster, Germany) using FEI TIA software (version 4.0). Fibril characteristics were analyzed with ImageJ software (v2.0).

### NMR Spectroscopy

NMR experiments were acquired at 600 MHz on a Bruker Avance III spectrometer equipped with a triple resonance TCI cryoprobe or on a Bruker Avance NEO spectrometer equipped with a cryoprobe Prodigy TCI. All NMR spectra were processed with Topspin 4.1.1 software (Bruker, Karlsruhe, Germany) and analyzed using NMRFAM-SPARKY. One-dimensional ^1^H spectra were acquired at 25 °C on samples dissolved in deuterated buffer (20 mM sodium phosphate at pH 7.4, 50 mM NaCl). A total of 8 transients were acquired over a spectral width of 9615 Hz and 32,768 complex points with a recycle delay of 4 s. Saturation transfer difference (STD) experiments were acquired at 600 MHz, with 8 scans at 25 °C. Tau^4RD^ aggregates (obtained after 48 h incubation) were extensively washed to eliminate any other species eventually present. Selective saturation of the protein at 0.4 ppm frequency was carried out with a 2 s pulse train (40 Gaussian-shaped pulses of 50 ms separated by 1 ms intervals, field strength of 90 Hz) included in the relaxation delay, and a 25 ms spin-lock was used to reduce the broad background protein signal. The STD spectrum was obtained by subtracting the on-resonance spectrum from the off-resonance spectrum. WaterLOGSY experiments were performed with a 180° inversion pulse applied to the water signal at ∼4.7 ppm using a Gaussian-shaped selective pulse of 7.5 ms. Each WaterLOGSY spectrum was acquired with 240 scans and a mixing time of 1.5 s. In all experiments, water suppression was obtained using the excitation sculpting pulse scheme. Experiments with samples containing only the free compounds were acquired as a reference to verify the binding.

### Tau^4RD^ Condensates in the Presence of Coffee, Caffeine, and Genistein

Tau^4RD^ was mixed with a small amount (1.2% of the total protein) of tau^4RD^ labeled with fluorescein isothiocyanate (FITC) or with Alexa Fluor 488 (Thermo Fisher Scientific) to report its liquid–liquid phase separation, as previously reported.^16^ Liquid–liquid phase separation of tau^4RD^ was induced using heparin, in the absence or presence of espresso coffee, caffeine, or genistein at different concentrations (35–280 μg/mL). Where indicated, coffee, caffeine, and genistein were added to already-formed droplets (after 5 min). In all samples, 35 μM protein in 20 mM sodium phosphate buffer, pH 6.0, 30 mM NaCl, and 5 mM DTT was mixed with 8.75 μM heparin. For phase separation imaging, 7 μL of the solution was spotted onto a microscope slide, covered with a circular coverslip, and sealed with nail polish. Condensate images were acquired on a Leica TCS SP5 AOBS microscope to visualize the formation of droplets over time. Image analysis was performed with FIJI ImageJ software (v2.0).

### Sample Preparation for Cellular Viability and Seeding-Based Aggregation Assays

Solutions containing the 100 μM tau^4RD^ protein (in 20 mM sodium phosphate buffer at pH 7.4, 50 mM NaCl, 1 mM DTT, and protease inhibitor with EDTA) were filtered and then incubated in the absence or presence of 50 or 400 μg/mL coffee extract in static conditions at 37 °C for 24 h. The protein and heparin were in a 4:1 molar ratio. After the incubation of tau^4RD^ in buffer or with coffee extracts, a centrifugation step was performed at 20,000*g* for 30 min to separate the fibrils as a pellet (Figure 7A). The sample obtained from tau^4RD^ incubated in the presence of 400 μg/mL coffee extract did not contain insoluble aggregates and therefore the first centrifugation did not produce pellets. The sample was further treated as described below. The solution was separated by filtration through a 100 kDa MWCO filter to isolate the monomeric forms (Figure 7A red frame, filtrate) from the higher molecular weight aggregates of tau^4RD^ (Figure 7A red frame, retentate). All samples were verified by sodium dodecyl sulfate–polyacrylamide gel electrophoresis (SDS–PAGE, Figure S12A).

### Seeding-Based Aggregation and Immunoblot Analysis

Nontumoral human embryonic kidney cell lines (HEK293) stably expressing human full-length tau P301L fused with GFP^17^ were cultured in DMEM high glucose (Aurogene) supplemented with 10% FBS (fetal bovine serum, Aurogene), antibiotics (100 U of penicillin/mL, and 100 U of streptomycin/mL), and 1% l-glutamine (Aurogene) at 37 °C, 5% CO_2_ in a humidified incubator. Once 70–80% confluence was reached, the cells were collected using trypsin, counted, and seeded for the experiments. HEK293 cells were treated with 5 μM tau^4RD^ fibrils obtained in the absence or presence of 50 μg/mL coffee extract (pellet) or with the filtrate and the retentate obtained as described from tau^4RD^ aggregated in the presence of 400 μg/mL coffee extract. Lipofectamine LTX at 0.5% was employed as a transfection agent. For immunoblot analysis, 100,000 cells/well were seeded in a 24-well plate. After treatment, the cells were first scraped into Triton lysis buffer (1% Triton X-100 in 50 mM Tris, 150 mM NaCl, pH 7.6) containing protease and phosphatase inhibitors and incubated on ice for 15 min. Lysates were centrifuged at 20,000*g* for 30 min at 4 °C. Supernatants were kept as the “Triton 1 fraction,” whereas the pellets were washed once in Triton lysis buffer, separated again with centrifugation, resuspended in SDS lysis buffer (1% SDS in 50 mM Tris, pH 7.6) at a volume that is 1/3 of the Triton lysis buffer, and heated for 15 min at 80 °C. After centrifugation at 20,000*g*, supernatants were kept as the “SDS fraction”. Protein concentrations of Triton 1 fractions were determined with the BCA assay. According to protein concentration in the Triton 1 soluble fraction (considered as the protein standard), a proper amount of the SDS fraction was separated by SDS–PAGE and probed with the TAU-5 antibody, specific for the human tau^FL^. Immunoreactive proteins were detected using the ECL prime western blotting detection reagents (Ge Healthcare) according to the manufacturer′s instructions.

### Cell Viability Assay

H4-APPswe neuroglioma cells (stably expressing the APP Swedish mutation) were a generous gift from Prof. Mario Buffelli. The cell line was cultured in a humidified atmosphere of 5% CO_2_ and passaged in a complete growth medium: Dulbecco′s modified Eagle medium (DMEM) high glucose (Aurogene) containing 10% fetal bovine serum (FBS, Aurogene) supplemented with antibiotics (1% penicillin/streptomycin) and 1% glutamine (Aurogene). Once 70–80% confluence was reached, the cells were collected using trypsin, washed, and counted. Cell viability after tau^4RD^ sample treatment was evaluated by the reduction of the tetrazolium salt MTT (1-(4,5-dimethylthiazol-2-yl)-3,5-diphenylformazan, thiazolyl blue formazan), following the manufacturer′s protocol. Briefly, 1000 H4-APPswe cells/well were seeded in their exponential growth phase in a flat-bottom 96-well plate and were incubated at 37 °C in a 5% CO_2_ incubator.

After 24 h, the cells were treated with tau^4RD^ samples (pellet or retentate at 5 μM) obtained as previously described. After 48 h of treatment, the cells were incubated with 0.5 mg/mL MTT for 3 h at 37 °C, and insoluble formazan crystals were dissolved in 200 μL of DMSO. The absorbance measurement at 560 nm was employed to evaluate the reduced MTT. Experiments were performed in triplicate on a Tecan Infinite M200 Pro Microplate Reader.

### Statistical Analysis

Statistical analysis was applied to cell viability data and TEM morphological data on fibrils. Any statistically significant difference between samples was determined using one-way ANOVA analysis of variance followed by Dunnett′s multiple comparison test to compare the means from sample groups against a control group. The significance threshold was set at *P* = 0.05.

For the cell viability assay, measurements were performed in triplicates.

For TEM analysis, 10–20 measurements (from different images) for each parameter were analyzed. All sets of samples comply with normal distribution for the D′Agostino and Pearson test. Standard deviation (SD) was homogeneous according to Brown–Forsythe and Bartlett′s tests.

For both analyses, *P* values were indicated as follows: * = 0.01–0.05, ** = 0.001–0.01, *** = 0.0001–0.001, and **** <0.0001.

## Results

### NMR Characterization of the Extract from Arabica and Robusta Coffee Beans

**Figure 2:**
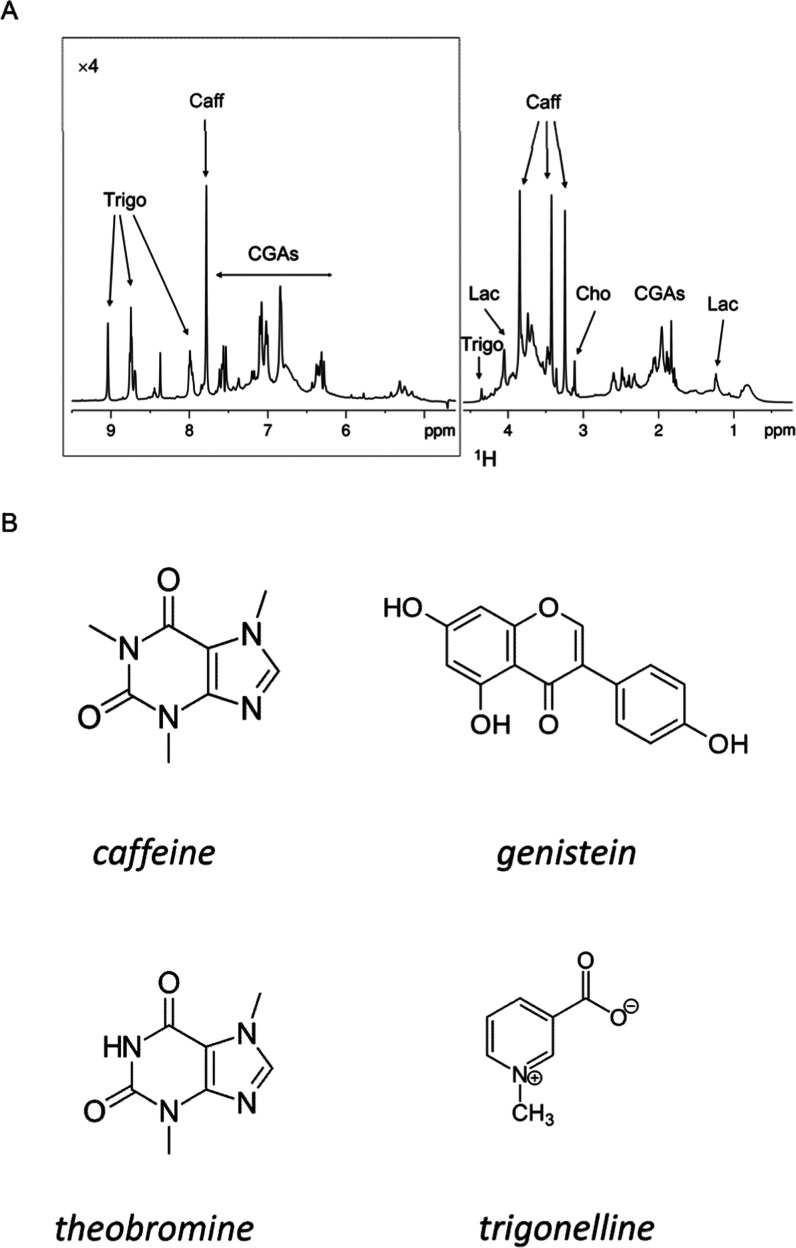
(A) ^1^H NMR profile of 5 mg/mL lyophilized espresso coffee extract. The downfield spectral region (5–10 ppm) is displayed with 4-fold higher intensity than the highfield region for better visualization. The spectrum has been recorded at 600 MHz and 25 °C. Peak assignments are indicated. Caff: caffeine, Trigo: trigonelline, CGAs: chlorogenic acids, Lac: lactate, and Cho: choline. (B) Molecular structures of coffee-derived molecules analyzed in this study.

Coffee samples were prepared with espresso extraction and examined by NMR spectroscopy in order to identify the main compounds constituting the beverage. The one-dimensional (1D) ^1^H NMR spectrum of espresso brew (Figure 2A) exhibits a complex profile in which the proton resonances are considerably overlapped. The identification of the main metabolites was obtained from the analysis of one- (^1^H) and two-dimensional (^1^H–^1^H TOCSY, ^1^H–^13^C HSQC) spectra and by comparison with previously reported data.^7,18,19^ The assignment of compounds such as trigonelline, caffeine, lactate, and chlorogenic acids (CGAs) was obtained from the observation of typical patterns of signals. The profiling of the coffee extract revealed the presence of compounds that are commonly found in brews from Arabica and Robusta coffee.^7^

### Coffee Extract and Single Compounds Influence the Aggregation Kinetics of Tau^4RD^

Following the identification of the main compounds contained in the coffee brew, we tested the impact of both the complex mixture and selected isolated compounds (Figure 2B) on protein tau fibril formation. We chose to focus on some compounds found in coffee extracts: two alkaloids, caffeine and trigonelline, and an isoflavone compound, genistein.^8,20,21^ Additionally, we employed the compound theobromine, a methylxanthine analogue to caffeine lacking the methyl group at position 1.

For aggregation experiments, we focused on a shorter construct of tau, hereafter tau^4RD^, spanning residues Q244-E372 (Figure 1). Tau^4RD^ comprises the microtubule-binding region and most of the residues involved in the assembly of pathological filaments,^22^ and it is widely used as a model system to test the aggregating properties of tau.^23,24^

**Figure 3:**
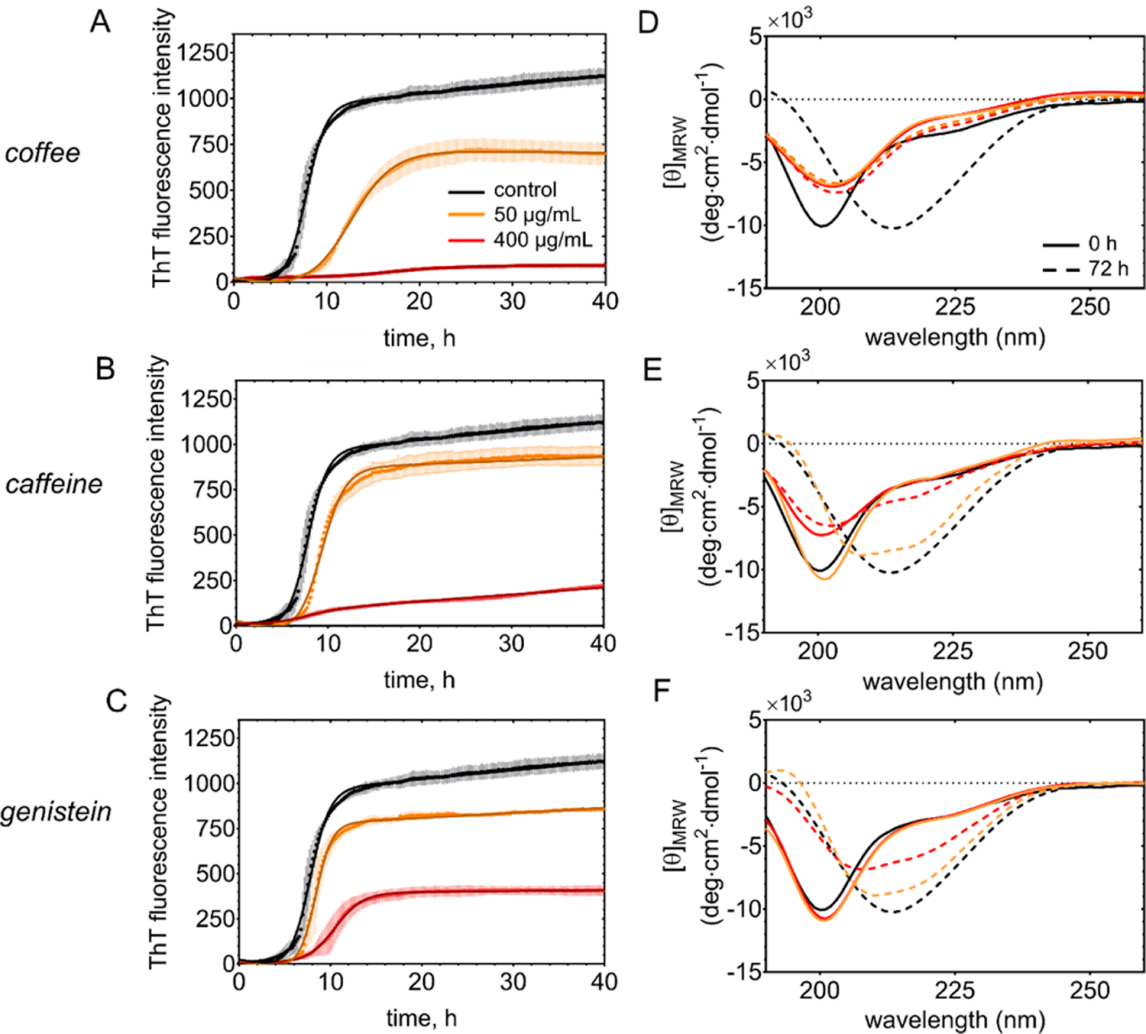
Time course of tau conformational transitions. (A–C) ThT fluorescence-based aggregation kinetics curves measured on 50 μM tau^4RD^ in the presence of coffee extract (A), caffeine (B), or genistein (C). Compound concentrations were 0 (black), 50 (orange), or 400 (red) μg/mL. Measurements were carried out on four replicates and data are reported as mean ± s.d. Solid lines correspond to the best-fit curves determined using an empirical sigmoid function. (D–F) Far-UV CD spectra recorded on 6 μM tau^4RD^ in the absence or presence of coffee compounds. Measurements were performed immediately after sample preparation (continuous curves) and after 72 h (dotted curves) incubation of a concentrated stock (50 μM protein and 50 or 400 μg/mL compounds) in static conditions at 37 °C. Molar concentrations of compounds were 0.26 mM (50 μg/mL) and 2 mM (400 μg/mL) caffeine and 0.18 mM (50 μg/mL) and 1.5 mM (400 μg/mL) genistein.

To assess the aggregation kinetics of tau^4RD^ in the presence and absence of coffee extract, we performed a ThT fluorescence assay. The fluorescence of ThT increases upon binding to β-sheet-rich amyloid structures and its change over time allows us to monitor fibril formation. In the presence of a low amount of coffee extract (50 μg/mL), the aggregation kinetics of tau^4RD^ followed a typical sigmoidal trend, comparable in shape to that observed for tau^4RD^ alone; however, it represents a significantly extended lag phase and a decreased rate of fibril growth (Figure 3A and Table 1). Interestingly, in the presence of 400 μg/mL coffee extract, there was almost no increase in ThT fluorescence and the typical sigmoidal shape was not discernable. These observations suggest an inhibitory effect of the coffee extract on tau fibril formation in a concentration-dependent manner (Figure 3A). This finding differs slightly from results described in a previous report,^25^ in which an effect on tau^FL^ fibrillization was observed only at a high concentration (200 μg/mL) of three different varieties of 100% Arabica instant coffee, while low concentrations (5 or 40 μg/mL) produced no or negligible variation of aggregation rates. We conclude that the specific coffee beans and the composition of the extracts determine a unique activity.

**Table 1:** Kinetic Parameters for the Aggregation of Tau^4RD^, Determined on the Basis of ThT Fluorescence Assays^a^

	concentration (μg/mL)	*t*_0.5_ (h)	τ (h)	*t*_lag_ (h)
control		7.85 ± 0.02	1.03 ± 0.02	5.79 ± 0.06
coffee	50	12.90 ± 0.06	2.09 ± 0.03	8.72 ± 0.12
	400	nd	nd	nd
caffeine	50	9.45 ± 0.03	1.17 ± 0.02	7.11 ± 0.07
	400	nd	nd	nd
genistein	50	8.43 ± 0.01	0.84 ± 0.01	6.75 ± 0.03
	400	10.50 ± 0.02	1.50 ± 0.01	7.50 ± 0.04
theobromine	50	9.10 ± 0.05	1.19 ± 0.04	6.72 ± 0.13
	400	8.46 ± 0.02	1.16 ± 0.02	6.14 ± 0.06
trigonelline	50	7.24 ± 0.02	1.00 ± 0.02	5.24 ± 0.06
	400	6.73 ± 0.01	0.56 ± 0.01	5.61 ± 0.03

a *t*_0.5_: midpoint of the transition; τ: elongation time constant; *t*_lag_ = *t*_0.5_ – 2τ; nd: not determined.

Next, we tried to determine if any of the components of the coffee extract were responsible for the inhibitory activity toward fibril formation. To this aim, we analyzed the aggregation kinetics of tau^4RD^ in the presence of increasing amounts of selected isolated molecules (Figures 3B,C and S1A,B). At a concentration of 50 μg/mL, the tested molecules showed moderate effects on fibril formation, with a consistently extended lag phase, except for trigonelline (Table 1). At a higher concentration (400 μg/mL), caffeine and genistein were found to strongly interfere with fibril formation. In particular, the ThT fluorescence response in the case of caffeine was very poor, similar to what was observed with the coffee extract (Figure 3A,B and Table 1). We also examined the effect of a mixture of the selected molecules at concentrations comparable to those estimated for the extract. The corresponding ThT fluorescence data indicate that the pool of compounds had some inhibitory effect on tau^4RD^ fibril formation, albeit not as strong as that of the coffee extract (Figure S1E). In order to exclude that the observed inhibitory effects were due to an interaction of the selected compounds with ThT (the fluorescence probe) or with heparin (the aggregation inducer), we acquired ^1^H NMR spectra of heparin or ThT in the absence and presence of different concentrations of each molecule (Figures S2–S5). The invariance of the signals of heparin or ThT, and of the compounds, indicated that no strong interactions occurred and that the effects observed during the fibrillization reactions originated from an interaction of the pure compounds with the protein substrate.

The described experiments indicate that the ensemble of compounds constituting the coffee extract is more effective in inhibiting tau^4RD^ fibril formation than any single component. Nonetheless, among the selected molecules, caffeine and genistein were capable to mitigate the process of tau^4RD^ aggregation.

### Conformational Transitions of Tau^4RD^ Analyzed by CD Spectroscopy

The maturation of amyloid fibers is triggered by the formation of aggregates characterized by a β-sheet secondary structure. To assess the effects of the coffee extract and of bioactive compounds on the conformational transitions of tau^4RD^ during aggregation, we acquired circular dichroism (CD) spectra and examined their overall shapes. Monomeric tau^4RD^ displays a far-UV CD spectrum typical of disordered polypeptides, characterized by a negative ellipticity peak centered at about 200 nm^23^ and the absence of strong signals at 220 nm (Figure 3D–F, black continuous line). This feature of the spectrum was maintained in all CD traces at the starting points of aggregation, independently of sample condition (Figure 3D–F), thus showing the inability of the compounds to modify the structure of soluble tau^4RD^. Changes in the secondary structure of tau^4RD^ were monitored after 72 h of incubation with heparin (Figure 3D–F, black dashed lines). The CD spectrum of the aggregated protein was characterized by a substantial change in shape and a shift of the curve minimum to longer wavelengths (∼215 nm), which is indicative of a structural reorganization consistent with the formation of β-sheet structures.

The latter behavior was retained in samples of tau^4RD^ coincubated with theobromine and trigonelline (Figure S1C,D), indicating that these compounds were unable to prevent aggregation. By contrast, a different behavior was observed when tau^4RD^ was incubated with genistein or caffeine, dependent on the concentration of the compounds (Figure 3E,F). At low molecule concentrations (Figure 3E,F orange lines), the conformational transformation of tau^4RD^ was affected to a small extent. At a higher concentration of genistein, we still observed a shift of the peak minimum but with smaller negative ellipticity. The impact of caffeine appeared even more pronounced: at 400 μg/mL concentration, the CD spectrum recorded after 72 h of incubation was only slightly altered from its initial shape (Figure 3E red lines), implying the prevalence of a disordered structure as a result of the inhibitory effect of caffeine on aggregation. Finally, the effect of coffee extract was quite strong, regardless of the concentration. The CD spectra were characterized by a reduced peak minimum suggestive of scattering effects in the samples and retained the initial shape after 72 h of incubation (Figure 3D). The conformational transitions of tau^4RD^ after 72 h of incubation with 50 μg/mL coffee extract (Figure 3D) were not detected by CD, possibly due to the complex composition of the sample and the heterogeneous nature of polymorphic nonfibrillar and fibrillar aggregates, which would not correspond to a unique secondary structure signature.

Overall, the results of CD experiments confirm that the compound mixture of coffee brew prevents the formation of ordered fibrillar structures. The conformational analysis agrees with the ThT analysis and indicates a variable impact on the aggregation properties of tau^4RD^ exerted by the coffee extract and by the single bioactive compounds.

### Morphological Analysis of Tau^4RD^ Aggregates

**Figure 4:**
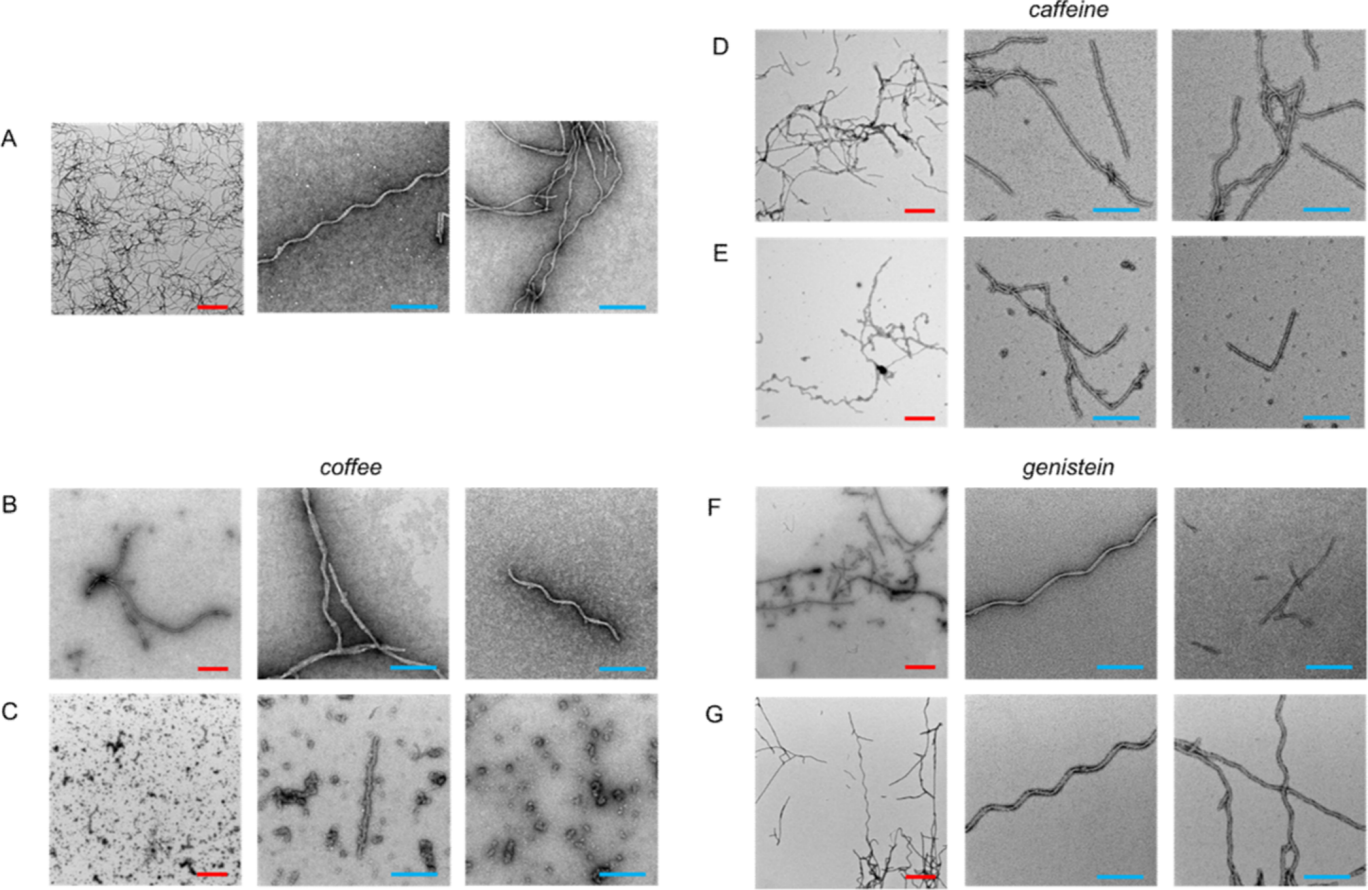
Transmission electron microscopy of tau^4RD^ aggregates. Representative TEM images of tau^4RD^ aggregates in the presence of different concentrations of coffee extract or single compounds. Samples contained tau^4RD^ in buffer (A) with 50 μg/mL (B) or 400 μg/mL (C) coffee extract, 50 μg/mL (D) or 400 μg/mL (E) caffeine, 50 μg/mL (F) or 400 μg/mL (G) genistein. The protein was 50 μM. Samples were incubated for 48 h at 37 °C in static conditions. Scale bars are 500 nm (red) and 200 nm (light blue).

As a next step, we performed a TEM analysis of tau^4RD^ aggregates obtained in the absence or presence of coffee extract or single compounds (Figures 4 and S6). The collected images clearly show that tau^4RD^ was able to form abundant mature fibrils after 48 h of incubation in the presence of heparin. Furthermore, TEM analysis confirmed the different abilities of the tested compounds to interfere with tau^4RD^ fibril formation. The presence of trigonelline and theobromine had a modest impact on tau^4RD^ fibril assembly, and long straight and twisted fibrils were observed at all tested concentrations (Figure S6); these data agree with aggregation kinetics results obtained by ThT fluorescence and with CD analysis. A different behavior was observed when caffeine and genistein were present during the fibrillization process (Figure 4D–G). Both molecules displayed significant inhibitory activity toward tau^4RD^ fibrillization, with a stronger effect at a higher compound concentration. In the presence of 50 μg/mL compounds, long fibrils could still form but short filaments were also visible. Upon increasing the amount of the compounds to 400 μg/mL, almost all structures visible in TEM images were short filaments with a morphology comparable to that of tau^4RD^ alone (Figure S7).

The effect of coffee extract was remarkable: even in the presence of low quantities of the mixture, the formation of long fibrils was compromised and only few short fibrils were visible (Figure 4B,C). The coffee extract at high concentrations strongly interfered with fibril formation: the few observable fibrils showed a modified morphology, and a large amount of spheroidal oligomeric species, with a diameter of about 20 nm, were observed in the images (Figures 4B,C and S7). The morphological analysis of all of the samples showed that the overall shape of fibrils of tau^4RD^ alone (Figure S7) was maintained also when the fibrils were formed in the presence of the selected molecules; the differences observed in some structural parameters are attributable to the different quality of images rather than to a real morphological difference. The results obtained using a coffee extract generally agree with previously reported data obtained on tau^FL^ in the presence of instant coffee brews;^25^^25^ however, no influence of caffeine on fibril growth was found in the previous study. The discrepancy could be due to the different tau constructs used in the two studies. It is worth noticing that another work^26^ provided evidence that full-length tau can interact with caffeine. The docking study indicated that the R2 and R4 repeats of the MBD of tau (Figure 1) are the most likely binding sites for caffeine, and localized surface plasmon resonance spectroscopy data revealed that the binding of caffeine molecules to tau significantly reduced the formation of tau–tau complexes. These results could explain our finding that caffeine inhibits fibril formation, as the assembly of a tau–tau complex is a prerequisite for protein aggregation. Moreover, the presence of spheroidal aggregates in TEM images of tau^4RD^ obtained in the presence of 400 μg/mL coffee extract or caffeine is in agreement with the decreased rate constants determined from ThT experiments. Altogether, the collected pieces of evidence suggest that fibril elongation and possibly secondary nucleation processes are inhibited in particular by caffeine and bioactive compounds of coffee extracts.

### Condensation-Linked Aggregation of Tau^4RD^

Accumulating evidence indicates that tau is able to form and participate in biomolecular condensates.^24,27,28^ Cellular biomolecular condensates often contain proteins and RNA, they are thought to form through liquid–liquid phase separation (LLPS) and exhibit liquid-like properties.^29^ LLPS has been recognized as one of the key organizing principles by which eukaryotic cells control molecular localization and biochemical reactions.^30^ However, condensates are metastable and may transition from liquid-like to gel or solid-like states.^30^ In particular, age-related changes and pathological insults may promote abnormal phase transitions.^31^ The observation that tau undergoes LLPS has stimulated efforts to understand whether condensation may be linked to pathological aggregation.^24,27,28^

**Figure 5:**
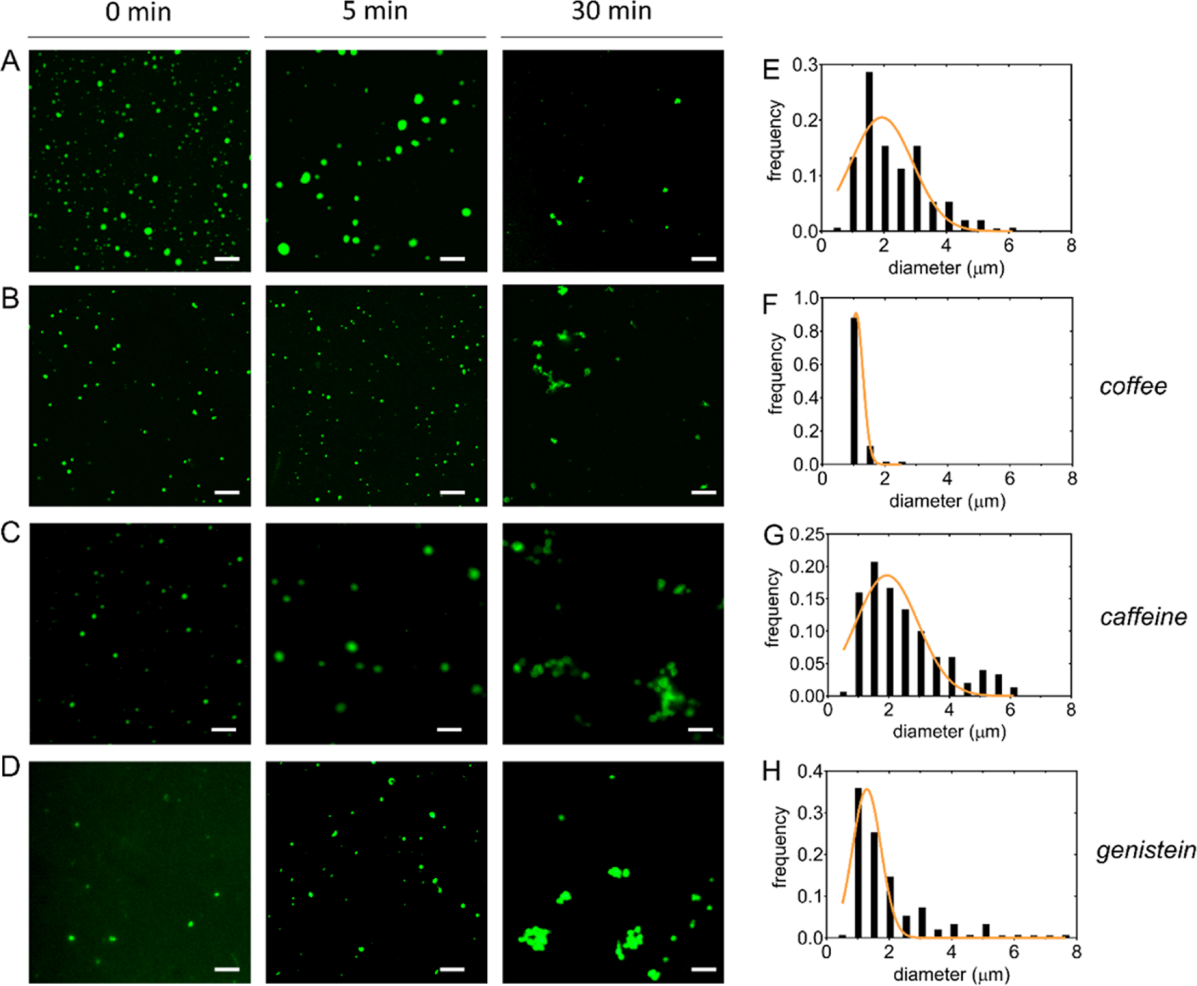
Influence of coffee compounds on tau condensation. Representative fluorescence microscopy images displaying condensates of tau^4RD^/heparin in simple buffer (A) or in the presence of 280 μg/mL coffee extract (B), caffeine (1.4 mM) (C), and genistein (1 mM) (D). Images were acquired at 0, 5, and 30 min after mixing components. Protein was 35 μM and heparin was 8.75 μM. The scale bar is 10 μm. (E–H) Distribution plots of droplet diameters corresponding to conditions of panels (A–D) at 5 min; orange lines are best-fit log-normal curves.

Polyanionic cofactors, including RNA and heparin, stimulate condensation of tau in vitro.^32^ Heparin is commonly used as an aggregation inducer, and it is increasingly employed in model systems to investigate condensation-linked aggregation.^16,33,34^ Indeed, heparin-induced liquid condensates of tau^4RD^ proved unstable and were found to rapidly evolve into irregularly shaped assemblies.^16,35^ Here, we prepared the condensates in the absence or presence of coffee extracts or single bioactive compounds and observed the liquid droplets by fluorescence microscopy using Alexa488-tau^4RD^ as a reporter molecule (Figures 5 and S8–S10). Small spherical droplets formed immediately after mixing tau^4RD^ and heparin, then grew over the following 5 min, and partly dissolved or lost their regular shape after 30 min (Figure 5A).

A similar evolution was observed in the presence of bioactive compounds; however, few differences emerged (Figure 5B–D): the size distribution of droplets in the presence of caffeine was similar to the control; by contrast, narrower distributions and smaller mean sizes were observed in the case of coffee extract and genistein. The endpoint (30 min) images displayed irregular assemblies for coffee and caffeine, while clustered spherical droplets appeared in the case of genistein. We further investigated if the bioactive compounds were able to perturb preformed condensates (Figures S8D,E, S9D,E, and S10D,E). We observed that condensates were minimally perturbed by the subsequent addition of compounds, with the exception of the case of 280 μg/mL genistein, which appeared to promote the coalescence of initially formed droplets into larger condensates.

In summary, the tested compounds did not dramatically impact the formation and evolution of tau^4RD^/heparin condensates with two main exceptions: coffee extracts prevented the formation of larger droplets, and concentrated genistein disfavored the aggregation-linked dispersion and transformation of droplets. This finding suggests that the coffee extract can influence the physicochemical properties of a condensate, modulating its stability and maturation, with important implications for disease-related condensation, an aspect that warrants further scrutiny.

### NMR-Based Assessment of the Binding of Coffee Extract and Caffeine to Tau^4RD^ Fibrils

The identification of soluble molecules able to interact with preformed fibrils is attracting increasing attention, as it opens new options for the design of novel therapeutics or specific probes for the diagnosis of a variety of neurodegenerative disorders.^36^ We therefore investigated the ability of coffee extract and caffeine to recognize and bind preformed tau fibrils by employing NMR spectroscopy, specifically a combination of STD^37^ and WaterLOGSY experiments.^38^ These experiments are quite powerful for assessing the interaction between a small molecule and a high-molecular-weight species. The latter are, in this case, the preformed tau fibrils dissolved in deuterated phosphate buffer. Both STD and WaterLOGSY are based on intermolecular nuclear Overhauser effects (NOEs) to the ^1^H nuclei of a ligand transiently bound to a large biomolecule.^39^

**Figure 6:**
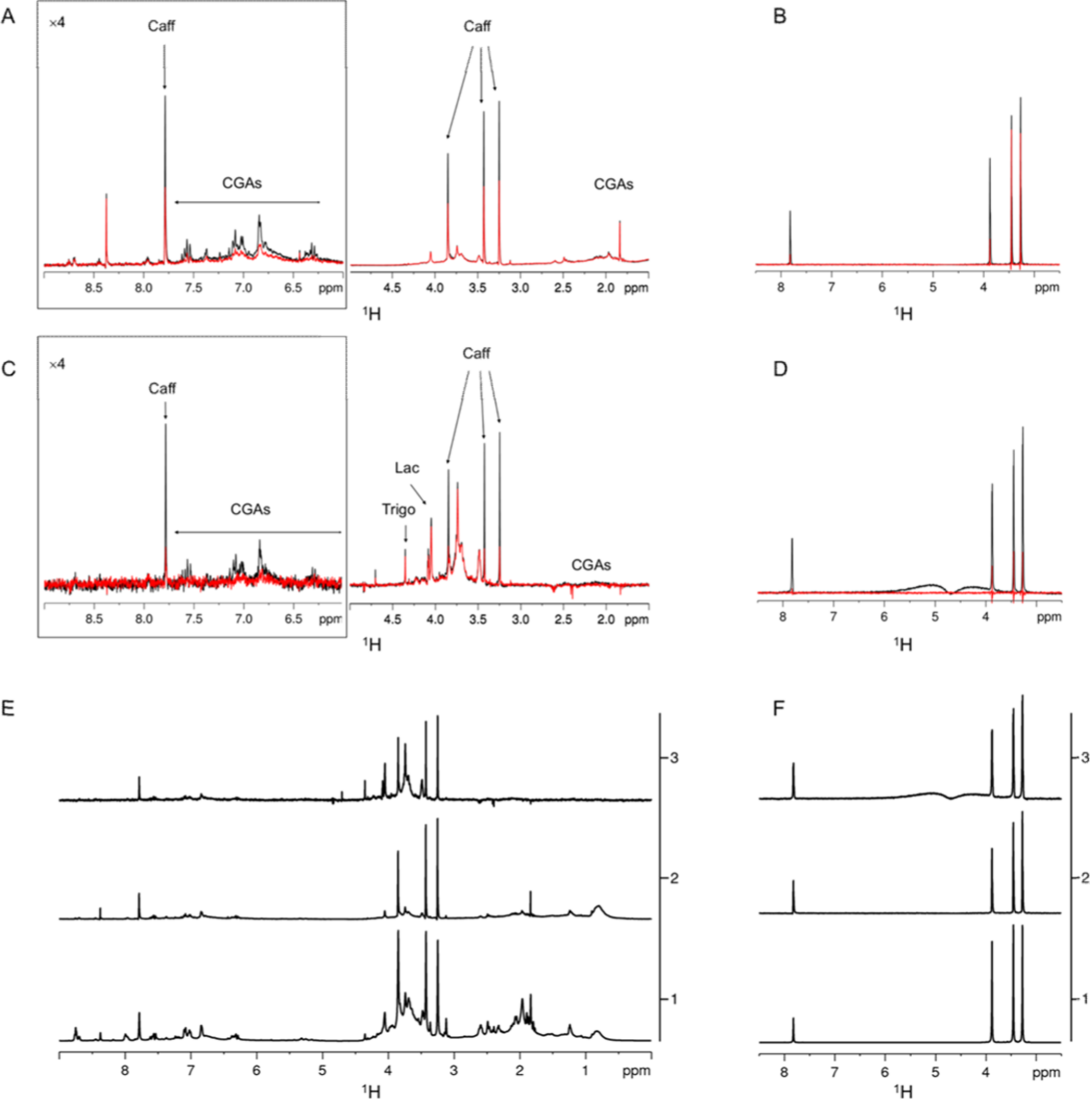
Interaction of coffee compounds with tau aggregates. Saturation transfer difference (A, B) and WaterLOGSY (C, D) NMR spectra acquired on a 5 mg/mL espresso coffee mixture (A, C) or 0.8 mg/mL (4 mM) caffeine (B, D) in the absence (red) and presence (black) of tau^4RD^ filaments (80 μM monomer) in 20 mM deuterated phosphate buffer, pH 7.4 at 25 °C. The downfield regions in (A, C) are displayed with four-fold higher intensity compared to the highfield regions on the right. (E, F) ^1^H NMR spectrum of 5 mg/mL coffee (E) or 0.8 mg/mL caffeine (F) in 20 mM deuterated phosphate buffer, pH 7.4 (1), STD (2), and WaterLOGSY (3) spectra acquired on 5 mg/mL coffee (E) or 0.8 mg/mL caffeine (F) in the presence of tau^4RD^ filaments.

The STD spectra of the coffee extract were acquired in the presence and absence (control experiment) of the protein fibrils, setting the on-resonance frequency at 0.4 ppm, to achieve saturation of protein aliphatic resonances. The signals in the STD spectrum (obtained by subtracting the on-resonance from the off-resonance spectrum) of the coffee extract contained several NMR signals (Figures 6A,E and S11A); however, the comparison with the control experiment acquired in the absence of fibrils revealed that only a subset of peaks experienced a signal build-up. Thus, a group of atoms of the molecules present in the coffee extract received saturation transfer from the protein via NOE, proving their interaction with the aggregates.^37^ We considered the results obtained with STD experiments definitive only if confirmed by WaterLOGSY experiments acquired in the same conditions (Figure 6C,E).^39,40^ In the case of WaterLOGSY, the ^1^H nuclei of bulk water are excited, and the magnetization is transferred from transiently bound water ^1^H to protons of a small molecule bound to the protein.^38^ Also, in the WaterLOGSY spectrum, a group of peaks experienced a signal build-up with respect to the control experiment. The comparison of the signals in STD and WaterLOGSY experiments with the ^1^H NMR spectrum of the coffee extract (Figure 6E) clearly indicates that caffeine and chlorogenic acids were able to interact with fibrils. To further prove the ability of caffeine to interact with tau^4RD^ fibrils, the STD and WaterLOGSY experiments were repeated on the pure compound in the same conditions (Figures 6B,D,F and S11B). All of the NMR peaks of caffeine experienced a signal build-up in the two experiments, thus confirming the ability of this bioactive molecule to bind to preformed tau^4RD^ fibrils.

Taken together, the NMR data provide clear evidence that specific bioactive molecules present in the coffee extract, i.e., caffeine and chlorogenic acids, can interact with preformed fibrils of tau^4RD^.

### Coffee Extract Modulates Tau-Mediated Cytotoxicity and Intracellular Tau Accumulation

**Figure 7:**
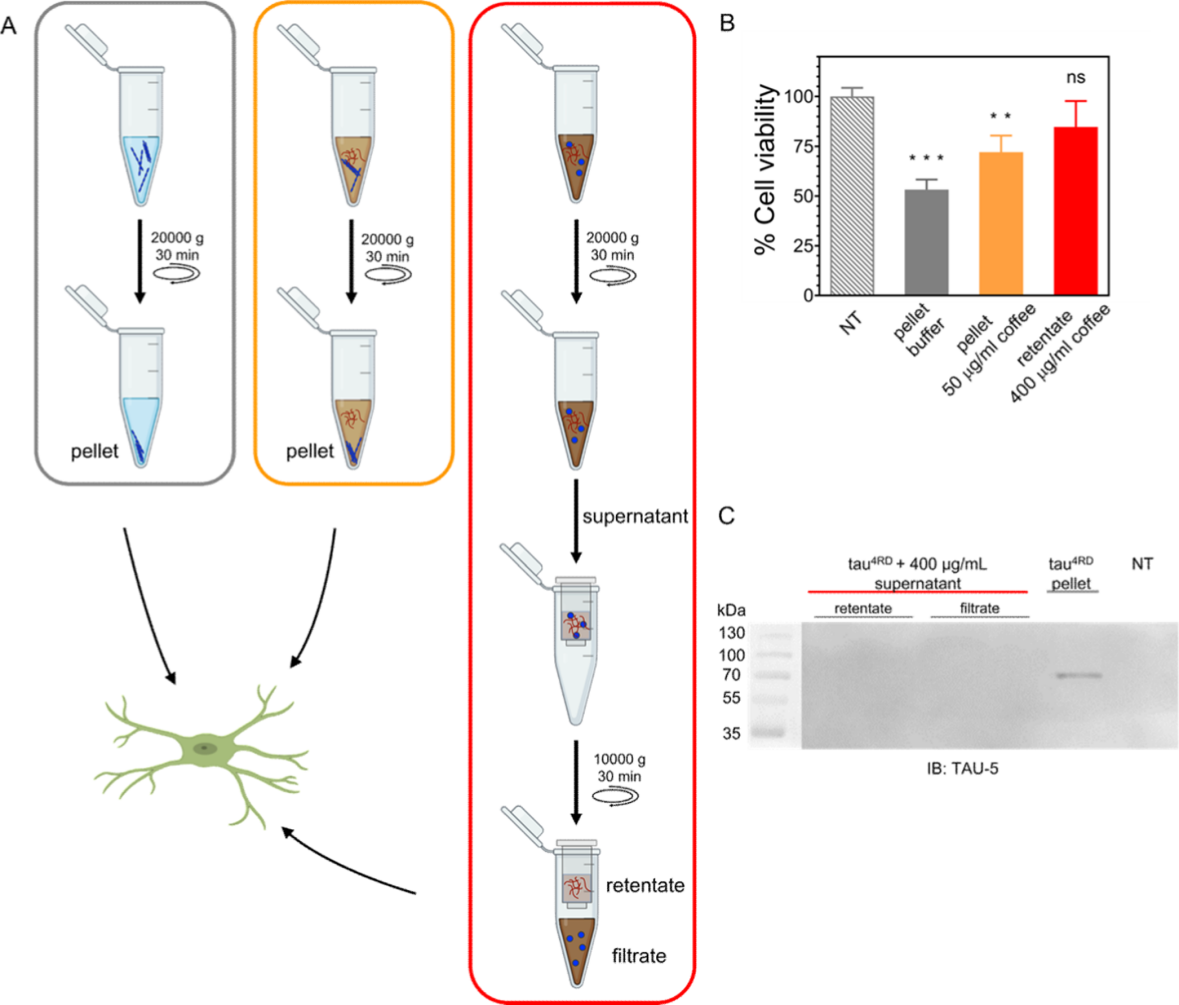
(A) Schematic depiction of sample preparation for cell viability and seeding-based aggregation assays in cellular models. A first centrifugation step was performed for the isolation of the fibrils (pellets in blue) from soluble tau (aggregates in dark red and monomers as blue spheres) obtained after the fibrillization reaction of tau^4RD^ in buffer (gray frame), in the presence of 50 μg/mL coffee extract (orange frame) and in the presence of 400 μg/mL coffee extract (red frame). The latter sample did not contain any aggregate and therefore the first centrifugation did not produce pellets. The solution obtained after centrifugation of tau^4RD^ aggregated in the presence of 400 μg/mL coffee extract was further treated: a filtration step (through 100 kDa MWCO) was performed to separate monomers (filtrate) from soluble aggregates (retentate) used for seeding-based aggregation assays. The figure was created with BioRender.com. (B) Cell viability assay performed on H4-APPswe neuroglioma cells nontreated (NT) or treated for 48 h with pellets or retentate obtained as depicted in A (the color code is maintained). One-way statistical analysis ANOVA followed by Dunnett′s multiple comparison test was performed, ns = nonsignificant, *p* = *0.01–0.05, **0.001–0.01, ***0.0001–0.001. (C) Immunoblot analysis of the cellular insoluble fraction after treatment with samples of tau^4RD^ aggregated in buffer and in the presence of 400 μg/mL coffee extract (prepared as depicted in A, red frame). HEK293 cells overexpressing tau^FL^/P310L-GFP were treated with the samples for 48 h and the Triton-insoluble fractions were blotted with the TAU-5 antibody. The last lane represents cells without the treatment (NT).

The above reported data point to the ability of the coffee extract to interfere with tau filament formation in vitro, redirecting the assembly of tau into off-pathway amorphous oligomeric species. We therefore decided to test the toxicity of tau^4RD^ aggregates formed in the absence and presence of different amounts of coffee extract. To this aim, neuroglioma-derived H4swe cells were treated for 48 h with three different samples obtained as depicted in Figure 7A. Fibrils were obtained by preincubating tau^4RD^ with heparin (4:1 ratio) and 50 μg/mL coffee extract and then sedimented by centrifugation (Figure 7A, orange frame, and S12A), showed decreased toxicity compared to fibrils obtained in simple buffer (Figure 7A, gray frame, and S12A), with cell viability increasing from 53 to 72% (100% refers to nontreated cells, Figure 7B).

We have shown that tau^4RD^ incubated in buffer containing 400 μg/mL coffee extract was unable to form fibrils but rather underwent structural transitions, giving rise to nonfibrillar aggregates/oligomeric species. Previous studies reported the ability of tau oligomers to act as primary culprits of toxicity;^41−43^ therefore, we sought to investigate if the amorphous, aggregated species formed in the presence of large quantities of coffee extract could interfere with cell survival. A sample containing tau^4RD^ aggregated species formed in the presence of 400 μg/mL coffee extract was first sedimented using centrifugation and checked on SDS–PAGE (Figure S12A). The soluble supernatant was then separated into monomers and mixtures of oligomers by filtration through a 100 kDa MWCO filter (Figures 7A red frame, and S12A). Interestingly, the viability of the cells treated with the retentate that contained amorphous/oligomeric species showed a nonsignificant difference with respect to the viability of nontreated cells (Figure 7B), thus indicating that the presence of coffee extract not only interferes with tau^4RD^ fibril formation but also promotes the formation of aggregated species with reduced or no toxicity.

Previous studies demonstrated the ability of synthetic preformed tau fibrils to act as a template in soluble tau fibrillization, both in vitro and in cell culture models.^17^ To test whether tau^4RD^ aggregates formed in the presence of coffee extract retained the seeding capacity, we employed HEK293 cells stably expressing tau^FL^/P301L-GFP and used lipofectamine to promote the cellular uptake of the selected samples. First, we treated the HEK293 tau^FL^/P301L-GFP cells with pellets isolated by centrifugation deriving from tau^4RD^ aggregated in the absence and presence of different amounts of coffee extract (sample preparation is depicted in Figure 7A). The immunoblot analysis of the Triton-insoluble fraction of cells after treatment with the tested samples showed that the seeded fibrillization propensity of tau^4RD^ was perturbed when the aggregates were obtained in the presence of coffee extract (Figure S12B). However, since the pellet of the sample obtained in the presence of 400 μg/mL coffee extract did not contain a detectable protein fraction (Figure S12A), we decided to treat the cells with the two components isolated by filtration of the soluble supernatant: the retentate and the filtrate (Figure 7A red frame). The immunoblot analysis confirmed the inability of the aggregates obtained in the presence of a high amount of coffee to induce intracellular tau fibrillization (Figure 7C). These data suggest that the molecules constituting the espresso coffee extract could have a bioactive role in the modulation of tau-mediated cell toxicity and reduce the tau-seeding activity in cultured cells.

## Discussion

Neurodegenerative diseases are often associated with the aggregation and deposition of incorrectly folded proteins. The predominant pathological feature of tauopathies, including Alzheimer′s disease, is the intraneuronal deposition of the tau protein, hence the development of novel forms of treatments and prevention targeting tau protein is considered a promising strategy.^44^ Toward this direction, a possible approach is to test small compounds for their inhibitory activity against the aggregation of tau.

In recent years, the potential health benefits of the consumption of functional foods have been extensively investigated,^45−48^ and a large number of food components showed biological activity.^49,50^ In this work, we investigated the antiaggregation property of espresso coffee extract and some of its components toward the tau protein. Taken together, ThT fluorescence, TEM analysis, and CD spectra indicate that the espresso extract has a strong antiaggregation effect in a dose-dependent manner. The length of tau^4RD^ fibrils decreased upon increasing coffee extract concentration and only small spheroidal oligomeric species were observed at a high coffee concentration.

We also found that the coffee extract can modulate the stability and maturation of tau condensates. Condensate formation has been suggested as a possible mechanism initiating the aggregation of tau;^27^ therefore, our results show a further interesting property of the coffee extract in interfering with the early events, leading to the pathological accumulation of tau.

Coffee extracts contain a large variety of bioactive compounds exhibiting health-beneficial effects.^2^ Using NMR-based analysis, we were able to identify the most abundant constituents of espresso coffee. Among these, we focused on caffeine and trigonelline, as well as on less abundant molecules, i.e., genistein and theobromine, to test their effects on tau^4RD^ aggregation. Among the tested compounds, only caffeine and genistein showed significant modulation of tau^4RD^ aggregation kinetics in a dose-dependent manner, and only short protofilaments were observed after incubation. Moreover, concentrated genistein modulates the transformation of droplets, suggesting a possible concerted bioactive function toward tau condensation and aggregation. It is interesting to point out that previous data showed an inhibitory activity of genistein against Aβ aggregation and toxicity.^50^ This dual inhibition function against tau and Aβ aggregation makes genistein an attractive biomolecule for designing genistein-based therapies.

It has been previously shown that caffeine, the well-known psychoactive alkaloid abundantly found in coffee extracts,^2^ does not prevent Aβ aggregation and neurotoxicity, therefore making this molecule less interesting as a bioactive compound.^8^ By contrast, our large panel of experiments clearly shows that caffeine exhibits a significant inhibitory activity toward tau^4RD^ aggregation. Furthermore, ligand-based NMR experiments showed that among all of the coffee components, caffeine has the additional property of binding preformed tau^4RD^ fibrils. All these findings are particularly interesting because caffeine could provide a structural template to treat tauopathies targeting tau fibrils or to design molecular probes with improved specificity and binding properties for the detection of pathological aggregates useful for the clinical diagnosis of tau-based diseases.

The formation of amorphous spheroidal aggregates in the presence of coffee extract prompted us to study their toxicity on H4-APPswe cells, as tau oligomers are considered to be the most toxic tau species. Tau^4RD^ aggregated in the presence of coffee extract showed a significantly decreased cytotoxicity compared with untreated tau^4RD^ fibrils, in line with previously reported data on natural compounds such as limonoids and xanthohumol.^51,52^ Additional experiments also showed the reduced ability of the amorphous aggregates to induce intracellular tau fibrillization, thus suggesting a neuroprotective effect of coffee extracts against tau-induced toxicity in cultured cells. These results are in line with previous studies showing a reduced seeding propensity of tau oligomers pretreated with a curcumin derivative.^53^

The association of coffee intake with a decreased risk of neurodegenerative disorders has been largely investigated.^2,54^ A moderate espresso coffee consumption of 2/3 cups per day (40 mL/cup) provides about 250 mg of caffeine and 25 μg of genistein, in addition to numerous other bioactive compounds.^7,20^ Many of these compounds, including caffeine and genistein, can cross the blood–brain barrier via different mechanisms^55,56^ and exert neuroprotective effects.^6^

Here, we show that aggregation of the tau protein is modulated by espresso coffee extract and some of its components, at both concentrations used in the experiments (i.e., 50 and 400 μg/mL). Intraneuronal tau concentration has been estimated to be about 2 μM,^57^ 25 times less than what we have used in our experiments. Based on the bioavailability of coffee components in the brain, and on the results of our study, we expect that moderate coffee consumption may provide a sufficient amount of bioactive molecules to act separately or synergistically as modulators of tau protein aggregation and toxicity.

In conclusion, we have presented a large body of evidence that espresso coffee, a widely consumed beverage, is a source of natural compounds showing beneficial properties in ameliorating tau-related pathologies. Our findings could pave the way for further investigation into the design of bioactive compounds in the prevention and treatment of tauopathies.

## Supporting Information Available

Time course of tau conformational transitions; interaction of compounds with heparin and ThT evaluated by NMR; aggregates morphology evaluated by TEM; quantitative analysis of aggregates morphology; influence of coffee extract on tau condensates; influence of caffeine on tau condensates; influence of genistein on tau condensates; interaction of coffee compounds with tau aggregates probed by NMR; and SDS–PAGE and immunoblot analysis of samples of in-cell experiments (PDF)

## Abbreviations

CD: circular dichroism
CGAs: chlorogenic acids
GFP: green fluorescent protein
HEK293: nontumoral human embryonic kidney cells
LLPS: liquid–liquid phase separation
MBD: microtubule-binding domain
MWCO: molecular weight cutoff
NMR: nuclear magnetic resonance
STD: saturation transfer difference
TEM: transmission electron microscopy
ThT: Thioflavin-T

## References

[ref1] Grosso G.; Godos J.; Galvano F.; Giovannucci E. L. Coffee, Caffeine, and Health Outcomes: An Umbrella Review. Annu. Rev. Nutr. 2017, 37, 131–156. 10.1146/annurev-nutr-071816-064941. 28826374

[ref2] Carneiro S. M.; Oliveira M. B. P. P.; Alves R. C. Neuroprotective properties of coffee: An update. Trends Food Sci. Technol. 2021, 113, 167–179. 10.1016/j.tifs.2021.04.052.

[ref3] Fan F. S. Coffee reduces the risk of hepatocellular carcinoma probably through inhibition of NLRP3 inflammasome activation by caffeine. Front. Oncol. 2022, 12, 1029491 10.3389/fonc.2022.1029491. 36330474 PMC9623052

[ref4] CarmanA. J.; DacksP. A.; LaneR. F.; ShinemanD. W.; FillitH. M. Current evidence for the use of coffee and caffeine to prevent age-related cognitive decline and Alzheimer′s disease. J. Nutr., Health Aging 2014, 18, 383–392. 10.1007/s12603-014-0021-7.24676319

[ref5] Santos C.; Costa J.; Santos J.; Vaz-Carneiro A.; Lunet N. Caffeine intake and dementia: systematic review and meta-analysis. J. Alzheimer’s Dis. 2010, 20, S187–204. 10.3233/JAD-2010-091387. 20182026

[ref6] Socała K.; Szopa A.; Serefko A.; Poleszak E.; Wlaz P. Neuroprotective Effects of Coffee Bioactive Compounds: A Review. Int. J. Mol. Sci. 2021, 22, 107 10.3390/ijms22010107. PMC7795778 33374338

[ref7] Ciaramelli C.; Palmioli A.; Airoldi C. Coffee variety, origin and extraction procedure: Implications for coffee beneficial effects on human health. Food Chem. 2019, 278, 47–55. 10.1016/j.foodchem.2018.11.063. 30583399

[ref8] Ciaramelli C.; Palmioli A.; De Luigi A.; Colombo L.; Sala G.; Riva C.; Zoia C. P.; Salmona M.; Airoldi C. NMR-driven identification of anti-amyloidogenic compounds in green and roasted coffee extracts. Food Chem. 2018, 252, 171–180. 10.1016/j.foodchem.2018.01.075. 29478529

[ref9] Zhang Y.; Wu K. M.; Yang L.; Dong Q.; Yu J. T. Tauopathies: new perspectives and challenges. Mol. Neurodegener. 2022, 17, 28 10.1186/s13024-022-00533-z. 35392986 PMC8991707

[ref10] Limorenko G.; Lashuel H. A. Revisiting the grammar of Tau aggregation and pathology formation: how new insights from brain pathology are shaping how we study and target Tauopathies. Chem. Soc. Rev. 2022, 51, 513–565. 10.1039/d1cs00127b. 34889934

[ref11] Wang Y.; Mandelkow E. Tau in physiology and pathology. Nat. Rev. Neurosci. 2016, 17, 5–21. 10.1038/nrn.2015.1. 26631930

[ref12] Barracchia C. G.; Tira R.; Parolini F.; Munari F.; Bubacco L.; Spyroulias G. A.; D’Onofrio M.; Assfalg M. Unsaturated Fatty Acid-Induced Conformational Transitions and Aggregation of the Repeat Domain of Tau. Molecules 2020, 25, 2716 10.3390/molecules25112716. 32545360 PMC7321374

[ref13] Ceccon A.; D’Onofrio M.; Zanzoni S.; Longo D. L.; Aime S.; Molinari H.; Assfalg M. NMR investigation of the equilibrium partitioning of a water-soluble bile salt protein carrier to phospholipid vesicles. Proteins 2013, 81, 1776–1791. 10.1002/prot.24329. 23760740

[ref14] Munari F.; Mollica L.; Valente C.; Parolini F.; Kachoie E. A.; Arrigoni G.; D’Onofrio M.; Capaldi S.; Assfalg M. Structural Basis for Chaperone-Independent Ubiquitination of Tau Protein by Its E3 Ligase CHIP. Angew. Chem., Int. Ed. 2022, 61, e202112374 10.1002/anie.202112374. PMC9303552 35107860

[ref15] Munari F.; Barracchia C. G.; Franchin C.; Parolini F.; Capaldi S.; Romeo A.; Bubacco L.; Assfalg M.; Arrigoni G.; D’Onofrio M. Semisynthetic and Enzyme-Mediated Conjugate Preparations Illuminate the Ubiquitination-Dependent Aggregation of Tau Protein. Angew. Chem., Int. Ed. 2020, 59, 6607–6611. 10.1002/anie.201916756. 32022419

[ref16] Parolini F.; Tira R.; Barracchia C. G.; Munari F.; Capaldi S.; D’Onofrio M.; Assfalg M. Ubiquitination of Alzheimer′s-related tau protein affects liquid-liquid phase separation in a site- and cofactor-dependent manner. Int. J. Biol. Macromol. 2022, 201, 173–181. 10.1016/j.ijbiomac.2021.12.191. 35016968

[ref17] Guo J. L.; Buist A.; Soares A.; Callaerts K.; Calafate S.; Stevenaert F.; Daniels J. P.; Zoll B. E.; Crowe A.; Brunden K. R.; et al. The Dynamics and Turnover of Tau Aggregates in Cultured Cells: INSIGHTS INTO THERAPIES FOR TAUOPATHIES. J. Biol. Chem. 2016, 291, 13175–13193. 10.1074/jbc.M115.712083. 27129267 PMC4933232

[ref18] Wei F. F.; Furihata K.; Hu F. Y.; Miyakawa T.; Tanokura M. Complex mixture analysis of organic compounds in green coffee bean extract by two-dimensional NMR spectroscopy. Magn. Reson. Chem. 2010, 48, 857–865. 10.1002/mrc.2678. 20818806

[ref19] D’Amelio N.; De Angelis E.; Navarini L.; Schievano E.; Mammi S. Green coffee oil analysis by high-resolution nuclear magnetic resonance spectroscopy. Talanta 2013, 110, 118–127. 10.1016/j.talanta.2013.02.024. 23618184

[ref20] Alves R. C.; Almeida I. M.; Casal S.; Oliveira M. B. Isoflavones in coffee: influence of species, roast degree, and brewing method. J. Agric. Food Chem. 2010, 58, 3002–3007. 10.1021/jf9039205. 20131840

[ref21] Angeloni S.; Navarini L.; Khamitova G.; Maggi F.; Sagratini G.; Vittori S.; Caprioli G. A new analytical method for the simultaneous quantification of isoflavones and lignans in 25 green coffee samples by HPLC-MS/MS. Food Chem. 2020, 325, 126924 10.1016/j.foodchem.2020.126924. 32387932

[ref22] Fitzpatrick A. W. P.; Falcon B.; He S.; Murzin A. G.; Murshudov G.; Garringer H. J.; Crowther R. A.; Ghetti B.; Goedert M.; Scheres S. H. W. Cryo-EM structures of tau filaments from Alzheimer′s disease. Nature 2017, 547, 185–190. 10.1038/nature23002. 28678775 PMC5552202

[ref23] Kumar S.; Tepper K.; Kaniyappan S.; Biernat J.; Wegmann S.; Mandelkow E. M.; Muller D. J.; Mandelkow E. Stages and conformations of the Tau repeat domain during aggregation and its effect on neuronal toxicity. J. Biol. Chem. 2014, 289, 20318–20332. 10.1074/jbc.M114.554725. 24825901 PMC4106345

[ref24] Ambadipudi S.; Biernat J.; Riedel D.; Mandelkow E.; Zweckstetter M. Liquid-liquid phase separation of the microtubule-binding repeats of the Alzheimer-related protein Tau. Nat. Commun. 2017, 8, 275 10.1038/s41467-017-00480-0. 28819146 PMC5561136

[ref25] Mancini R. S.; Wang Y.; Weaver D. F. Phenylindanes in Brewed Coffee Inhibit Amyloid-Beta and Tau Aggregation. Front. Neurosci. 2018, 12, 735 10.3389/fnins.2018.00735. 30369868 PMC6194148

[ref26] Yekta R.; Sadeghi L.; Ahmadi-Kandjani S.; Naziri P.; Rashidi M. R.; Dehghan G. The impact of caffeine on tau-tau interaction: LSPR detection, structural modification and molecular dynamics simulation. J. Mol. Liq. 2021, 338, 115914 10.1016/j.molliq.2021.115914.

[ref27] Wegmann S.; Eftekharzadeh B.; Tepper K.; Zoltowska K. M.; Bennett R. E.; Dujardin S.; Laskowski P. R.; MacKenzie D.; Kamath T.; Commins C.; et al. Tau protein liquid-liquid phase separation can initiate tau aggregation. EMBO J. 2018, 37, e98049 10.15252/embj.201798049. 29472250 PMC5881631

[ref28] Boyko S.; Surewicz K.; Surewicz W. K. Regulatory mechanisms of tau protein fibrillation under the conditions of liquid-liquid phase separation. Proc. Natl. Acad. Sci. U.S.A. 2020, 117, 31882–31890. 10.1073/pnas.2012460117. 33262278 PMC7749306

[ref29] Uversky V. N. Intrinsically disordered proteins in overcrowded milieu: Membrane-less organelles, phase separation, and intrinsic disorder. Curr. Opin. Struct. Biol. 2017, 44, 18–30. 10.1016/j.sbi.2016.10.015. 27838525

[ref30] Alberti S.; Gladfelter A.; Mittag T. Considerations and Challenges in Studying Liquid-Liquid Phase Separation and Biomolecular Condensates. Cell 2019, 176, 419–434. 10.1016/j.cell.2018.12.035. 30682370 PMC6445271

[ref31] Shin Y.; Brangwynne C. P. Liquid phase condensation in cell physiology and disease. Science 2017, 357, eaaf4382 10.1126/science.aaf4382. 28935776

[ref32] Zhang X.; Lin Y.; Eschmann N. A.; Zhou H.; Rauch J. N.; Hernandez I.; Guzman E.; Kosik K. S.; Han S. RNA stores tau reversibly in complex coacervates. PLoS Biol. 2017, 15, e2002183 10.1371/journal.pbio.2002183. 28683104 PMC5500003

[ref33] Lin Y.; McCarty J.; Rauch J. N.; Delaney K. T.; Kosik K. S.; Fredrickson G. H.; Shea J. E.; Han S. Narrow equilibrium window for complex coacervation of tau and RNA under cellular conditions. eLife 2019, 8, e42571 10.7554/eLife.42571. 30950394 PMC6450672

[ref34] Ukmar-Godec T.; Hutten S.; Grieshop M. P.; Rezaei-Ghaleh N.; Cima-Omori M. S.; Biernat J.; Mandelkow E.; Soding J.; Dormann D.; Zweckstetter M. Lysine/RNA-interactions drive and regulate biomolecular condensation. Nat. Commun. 2019, 10, 2909 10.1038/s41467-019-10792-y. 31266957 PMC6606616

[ref35] Boyko S.; Surewicz W. K. Tau liquid-liquid phase separation in neurodegenerative diseases. Trends Cell Biol. 2022, 32, 611–623. 10.1016/j.tcb.2022.01.011. 35181198 PMC9189016

[ref36] Buell A. K.; Esbjorner E. K.; Riss P. J.; White D. A.; Aigbirhio F. I.; Toth G.; Welland M. E.; Dobson C. M.; Knowles T. P. Probing small molecule binding to amyloid fibrils. Phys. Chem. Chem. Phys. 2011, 13, 20044–20052. 10.1039/c1cp22283j. 22006124

[ref37] Mayer M.; Meyer B. Characterization of Ligand Binding by Saturation Transfer Difference NMR Spectroscopy. Angew. Chem., Int. Ed. 1999, 38, 1784–1788. 10.1002/(SICI)1521-3773(19990614)38:12<1784::AID-ANIE1784>3.0.CO;2-Q. 29711196

[ref38] Dalvit C.; Fogliatto G.; Stewart A.; Veronesi M.; Stockman B. WaterLOGSY as a method for primary NMR screening: practical aspects and range of applicability. J. Biomol. NMR 2001, 21, 349–359. 10.1023/a:1013302231549. 11824754

[ref39] Antanasijevic A.; Ramirez B.; Caffrey M. Comparison of the sensitivities of WaterLOGSY and saturation transfer difference NMR experiments. J. Biomol. NMR 2014, 60, 37–44. 10.1007/s10858-014-9848-9. 25015532 PMC4201884

[ref40] Ferrari F.; Bissaro M.; Fabbian S.; De Almeida Roger J.; Mammi S.; Moro S.; Bellanda M.; Sturlese M. HT-SuMD: making molecular dynamics simulations suitable for fragment-based screening. A comparative study with NMR. J. Enzyme Inhib. Med. Chem. 2021, 36, 1–14. 10.1080/14756366.2020.1838499. 33115279 PMC7598995

[ref41] Kayed R.; Head E.; Thompson J. L.; McIntire T. M.; Milton S. C.; Cotman C. W.; Glabe C. G. Common structure of soluble amyloid oligomers implies common mechanism of pathogenesis. Science 2003, 300, 486–489. 10.1126/science.1079469. 12702875

[ref42] Gibbons G. S.; Lee V. M. Y.; Trojanowski J. Q. Mechanisms of Cell-to-Cell Transmission of Pathological Tau: A Review. JAMA Neurol. 2019, 76, 101–108. 10.1001/jamaneurol.2018.2505. 30193298 PMC6382549

[ref43] Ghag G.; Bhatt N.; Cantu D. V.; Guerrero-Munoz M. J.; Ellsworth A.; Sengupta U.; Kayed R. Soluble tau aggregates, not large fibrils, are the toxic species that display seeding and cross-seeding behavior. Protein Sci. 2018, 27, 1901–1909. 10.1002/pro.3499. 30125425 PMC6201727

[ref44] Soeda Y.; Takashima A. New Insights Into Drug Discovery Targeting Tau Protein. Front. Mol. Neurosci. 2020, 13, 590896 10.3389/fnmol.2020.590896. 33343298 PMC7744460

[ref45] Hewlings S. J.; Kalman D. S. Curcumin: A Review of Its Effects on Human Health. Foods 2017, 6, 92 10.3390/foods6100092. 29065496 PMC5664031

[ref46] Nocella C.; Cammisotto V.; Fianchini L.; D’Amico A.; Novo M.; Castellani V.; Stefanini L.; Violi F.; Carnevale R. Extra Virgin Olive Oil and Cardiovascular Diseases: Benefits for Human Health. Endocr., Metab. Immune Disord.: Drug Targets 2017, 18, 4–13. 10.2174/1871530317666171114121533. 29141571

[ref47] Markellos C.; Ourailidou M. E.; Gavriatopoulou M.; Halvatsiotis P.; Sergentanis T. N.; Psaltopoulou T. Olive oil intake and cancer risk: A systematic review and meta-analysis. PLoS One 2022, 17, e0261649 10.1371/journal.pone.0261649. 35015763 PMC8751986

[ref48] Hinojosa-Nogueira D.; Perez-Burillo S.; de la Cueva S. P.; Rufian-Henares J. A. Green and white teas as health-promoting foods. Food Funct. 2021, 12, 3799–3819. 10.1039/d1fo00261a. 33977999

[ref49] Ciaramelli C.; Palmioli A.; De Luigi A.; Colombo L.; Sala G.; Salmona M.; Airoldi C. NMR-based Lavado cocoa chemical characterization and comparison with fermented cocoa varieties: Insights on cocoa′s anti-amyloidogenic activity. Food Chem. 2021, 341, 128249 10.1016/j.foodchem.2020.128249. 33038804

[ref50] Ren B.; Liu Y.; Zhang Y.; Cai Y.; Gong X.; Chang Y.; Xu L.; Zheng J. Genistein: A Dual Inhibitor of Both Amyloid beta and Human Islet Amylin Peptides. ACS Chem. Neurosci. 2018, 9, 1215–1224. 10.1021/acschemneuro.8b00039. 29432676

[ref51] Zhang M.; Wu Q.; Yao X.; Zhao J.; Zhong W.; Liu Q.; Xiao S. Xanthohumol inhibits tau protein aggregation and protects cells against tau aggregates. Food Funct. 2019, 10, 7865–7874. 10.1039/c9fo02133g. 31793596

[ref52] Gorantla N. V.; Das R.; Mulani F. A.; Thulasiram H. V.; Chinnathambi S. Neem Derivatives Inhibits Tau Aggregation. J. Alzheimer Dis. Rep. 2019, 3, 169–178. 10.3233/ADR-190118. PMC6597962 31259310

[ref53] Lo Cascio F.; Puangmalai N.; Ellsworth A.; Bucchieri F.; Pace A.; Piccionello A. P.; Kayed R. Toxic Tau Oligomers Modulated by Novel Curcumin Derivatives. Sci. Rep. 2019, 9, 19011 10.1038/s41598-019-55419-w. 31831807 PMC6908736

[ref54] Ross G. W.; Abbott R. D.; Petrovitch H.; Morens D. M.; Grandinetti A.; Tung K. H.; Tanner C. M.; Masaki K. H.; Blanchette P. L.; Curb J. D.; et al. Association of coffee and caffeine intake with the risk of Parkinson disease. JAMA 2000, 283, 2674–2679. 10.1001/jama.283.20.2674. 10819950

[ref55] Tsai T. H. Concurrent measurement of unbound genistein in the blood, brain and bile of anesthetized rats using microdialysis and its pharmacokinetic application. J. Chromatogr. A 2005, 1073, 317–322. 10.1016/j.chroma.2004.10.048. 15909536

[ref56] McCall A. L.; Millington W. R.; Wurtman R. J. Blood-brain barrier transport of caffeine: dose-related restriction of adenine transport. Life Sci. 1982, 31, 2709–2715. 10.1016/0024-3205(82)90715-9. 7154859

[ref57] Iqbal K.; Liu F.; Gong C. X.; Grundke-Iqbal I. Tau in Alzheimer disease and related tauopathies. Curr. Alzheimer Res. 2010, 7, 656–664. 10.2174/156720510793611592. 20678074 PMC3090074

