# Supplementary material for: Espresso Coffee Mitigates the Aggregation and Condensation of Alzheimer′s Associated Tau Protein
Source: J Agric Food Chem. 2023 Jul 19;71(30):11429–41. doi: 10.1021/acs.jafc.3c01072 (PMC10401709; doi:10.1021/acs.jafc.3c01072)
Supplement: Supplementary file 1 — jf3c01072_si_001.pdf [file jf3c01072_si_001.pdf]

# *Supporting information*

**Espresso coffee mitigates the aggregation and condensation of Alzheimer's associated tau protein**

Roberto Tira[1], Giovanna Viola[1], Carlo Giorgio Barracchia[1], Francesca Parolini[1], Francesca Munari[1], Stefano Capaldi[1], Michael Assfalg[1], Mariapina D'Onofrio[1*].

[1]Department of Biotechnology, University of Verona, Strada le Grazie 15, 34134 Verona, Italy
*Corresponding author at: Department of Biotechnology, University of Verona, Strada le Grazie 15, 34134 Verona, Italy
Phone number: +39 0458027801
E-mail address: mariapina.donofrio@univr.it

This PDF file includes
Figures S1-S12

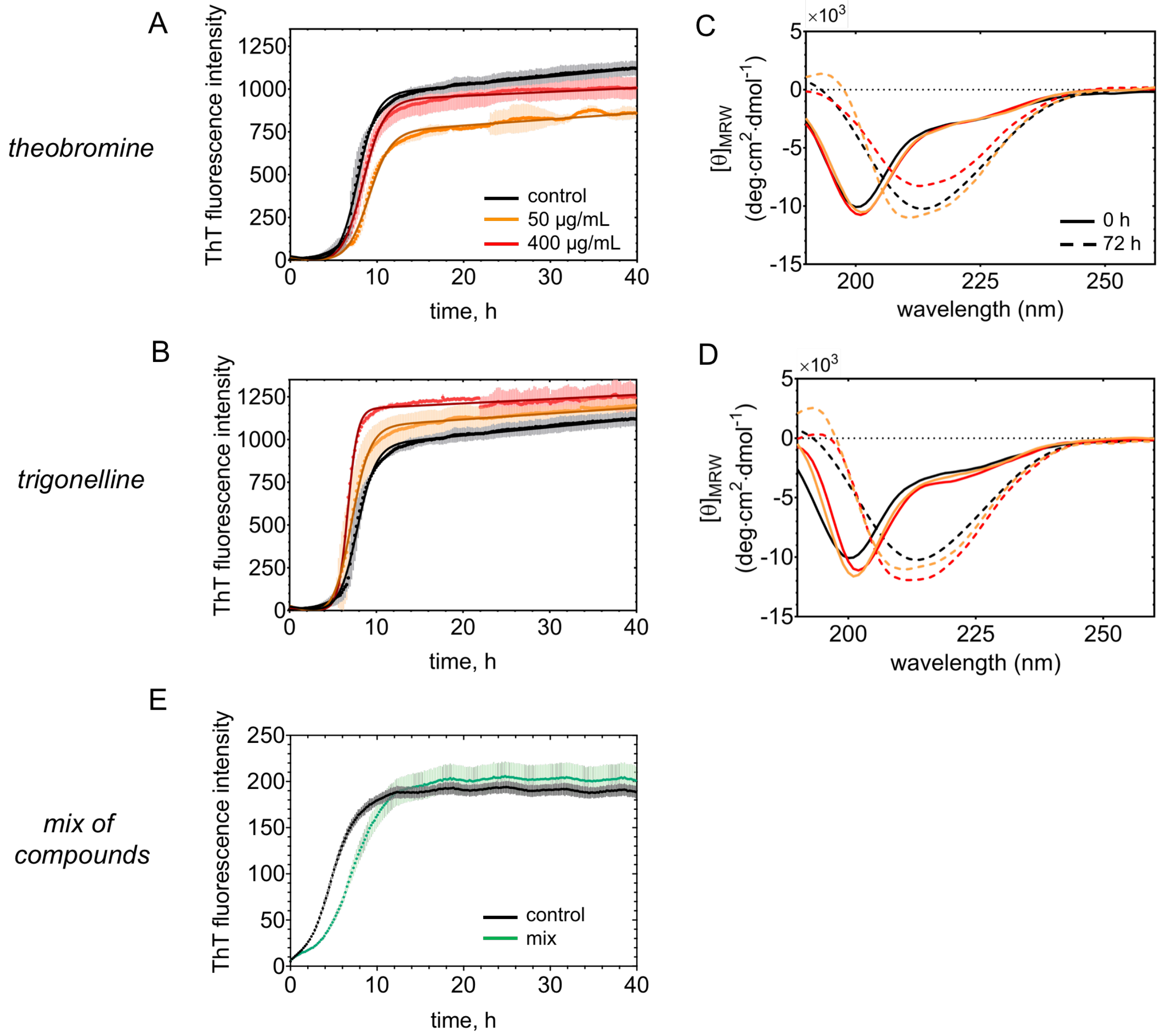


**Figure S1.** *Time course of tau conformational transitions*. (A,B) ThT fluorescence-based aggregation kinetics curves measured on 50 μM tau$^{4RD}$ in the presence of (A) theobromine, (B) trigonelline. Compound concentrations were 0 (black), 50 (orange) or 400 (red) μg/mL. Molar concentrations of compounds were: 0.30 mM (50 μg/mL) and 2.2 mM (400 μg/mL) theobromine; 0.29 mM (50 μg/mL) and 2.3 mM (400 μg/mL) trigonelline. (E) ThT fluorescence-based aggregation kinetics curves measured on 50 μM tau$^{4RD}$ in the presence of a mixture of compounds prepared as follows: 10 μg/mL (50 μM) caffeine, 1 μg/mL (3.7 μM) genistein, 1 μg/mL (5.5 μM) theobromine, 5 μg/mL (36 μM) trigonelline. Measurements were carried out on four replicates and data are reported as mean ± s.d.. Solid lines correspond to the best fit curves determined using an empirical sigmoid function. (C, D) Far-UV CD spectra recorded on 6 μM tau$^{4RD}$ in the presence of (C) theobromine, (D) trigonelline. Measurements were performed immediately after sample preparation (continuous curves) and after 72 h (dotted curves) incubation of a concentrated stock (50 μM protein and 50 or 400 μg/mL compounds) in static conditions.

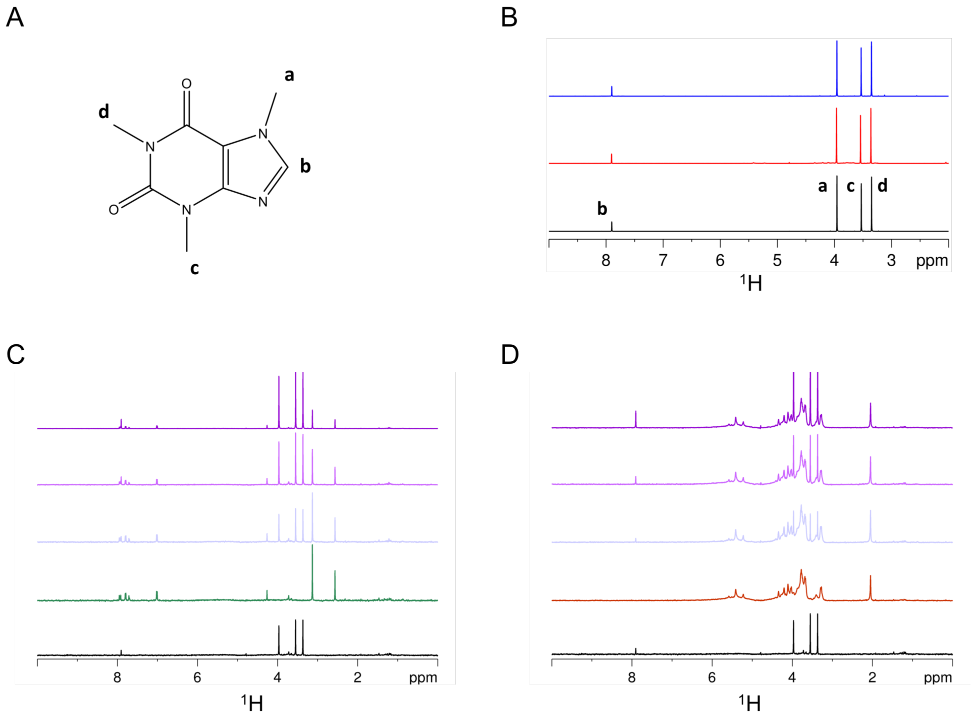


**Figure S2.** *Interaction of caffeine with heparin and with ThT evaluated by NMR.* (A) The chemical structure of caffeine with protons indications. (B) $^{1}$H NMR spectra of 400 µg/mL (2 mM) caffeine, in the absence (black) and in the presence of 50 µM heparin (red) or 50 µM thioflavin-T (blue) (molar ratios 40:1). Peaks assignments are shown. Spectra intensities have been adjusted for better visualization. (C) $^{1}$H NMR titration experiments of 50 µM ThT in the absence (green) or in the presence of caffeine at concentrations of 50 µM (lavender), 100 µM (purple), and 250 µM (violet). (D) $^{1}$H NMR titration experiments of 50 µM heparin in the absence (brown) or in the presence of caffeine at concentration of 50 µM (lavender), 100 µM (purple), and 250 µM (violet). The spectrum of the caffeine alone is reported as a reference (black). All the spectra were acquired at 600 MHz and 25 °C.

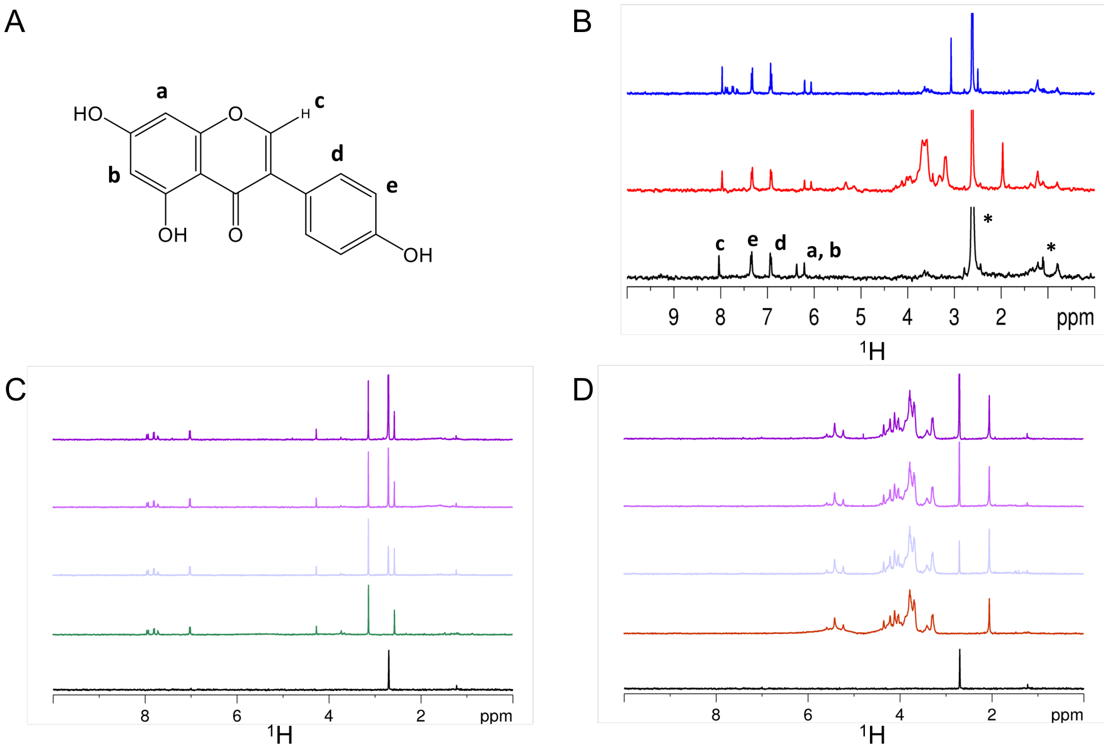


**Figure S3.** *Interaction of genistein with heparin and with ThT evaluated by NMR.* (A) The chemical structure of genistein with protons indications. (B) $^{1}$H NMR spectra of 400 µg/mL (1.5 mM) genistein, in the absence (black) and in the presence of 50 µM heparin (red) or 50 µM thioflavin-T (blue) (molar ratios 30:1). Peaks assignments are shown, * indicates impurities. Spectra intensities have been adjusted for better visualization. (C) $^{1}$H NMR titration experiments of 50 µM ThT in the absence (green) or in the presence of genistein at concentration of 50 µM (lavender), 100 µM (purple), and 250 µM (violet). (D) $^{1}$H NMR titration experiments of 50 µM heparin in the absence (brown) or in the presence of genistein at concentration of 50 µM (lavender), 100 µM (purple), and 250 µM (violet). The spectrum of the genistein alone is reported as a reference (black). All the spectra were acquired at 600 MHz and 25 °C.

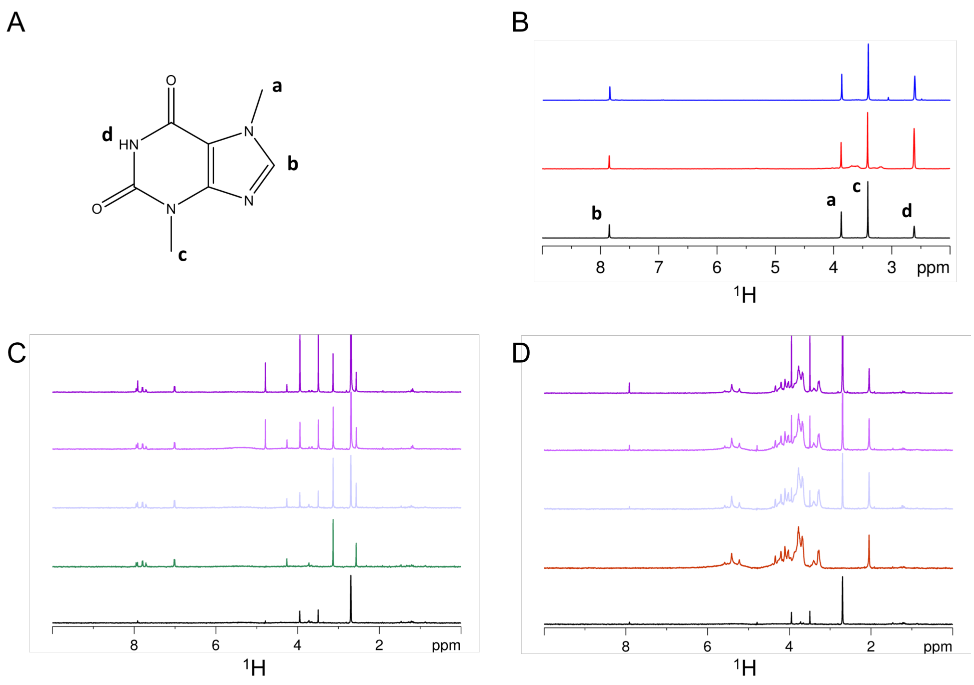


**Figure S4.** *Interaction of theobromine with heparin and with ThT evaluated by NMR.* (A) The chemical structure of theobromine with protons indications. (B) $^1$H NMR spectra of 400 µg/mL (2.2 mM) theobromine, in the absence (black) and in the presence of 50 µM heparin (red) or 50 µM thioflavin-T (blue) (molar ratios 44:1). Peaks assignments are shown. Spectra intensities have been adjusted for better visualization. (C) $^1$H NMR titration experiments of 50 µM ThT in the absence (green) or in the presence of theobromine at concentration of 50 µM (lavender), 100 µM (purple), and 250 µM (violet). (D) $^1$H NMR titration experiments of 50 µM heparin in the absence (brown) or in the presence of theobromine at concentration of 50 µM (lavender), 100 µM (purple), and 250 µM (violet). The spectrum of the theobromine alone is reported as a reference (black). All the spectra were acquired at 600 MHz and 25 °C.

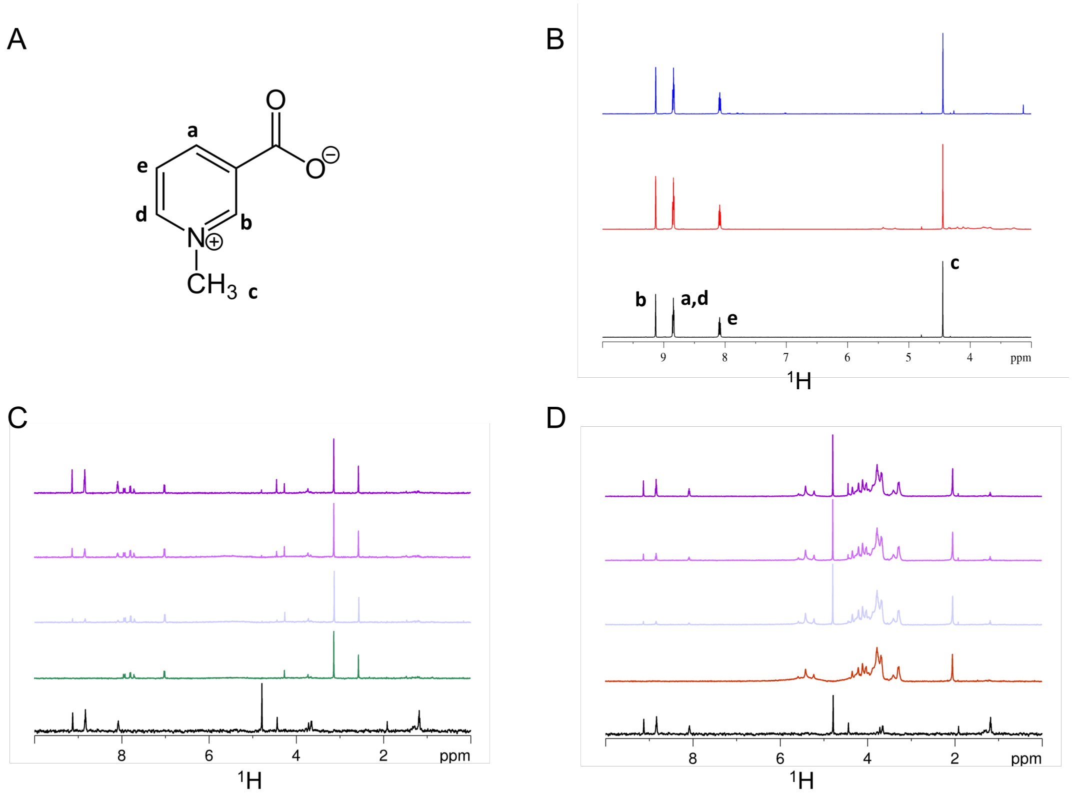


**Figure S5.** *Interaction of trigonelline with heparin and with ThT evaluated by NMR.* (A) The chemical structure of trigonelline with protons indications. (B) $^{1}H$ NMR spectra of 400 µg/mL (2.3 mM) trigonelline, in the absence (black) and in the presence of 50 µM heparin (red) or 50 µM thioflavin-T (blue) (molar ratios 46:1). Peaks assignments are shown. Spectra intensities have been adjusted for better visualization. (C) $^{1}H$ NMR titration experiments of 50 µM ThT in the absence (green) or in the presence of trigonelline at concentration of 50 µM (lavender), 100 µM (purple), and 250 µM (violet). (D) $^{1}H$ NMR titration experiments of 50 µM heparin in the absence (brown) or in the presence of trigonelline at concentration of 50 µM (lavender), 100 µM (purple), and 250 µM (violet). The spectrum of the trigonelline alone is reported as a reference (black). All the spectra were acquired at 600 MHz and 25 °C.

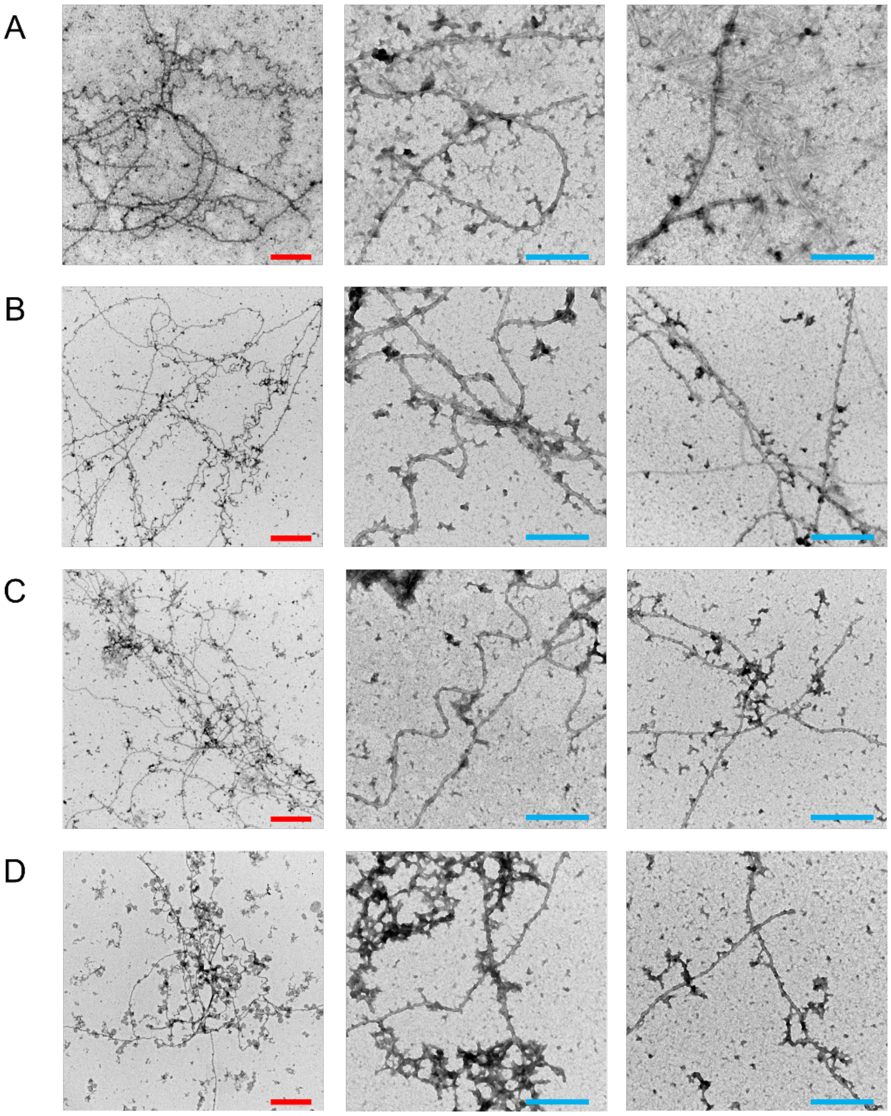


**Figure S6**. *Aggregates morphology evaluated by TEM*. TEM images of tau$^{4RD}$ filaments formed in the presence of bioactive compounds. Samples contained 50 μM tau$^{4RD}$ and 50 μg/mL (A) or 400 μg/mL (B) theobromine, 50 μg/mL (C), or 400 μg/mL (D) trigonelline. Samples were incubated for 48 h at 37 °C in static conditions. Scale bars are 500 nm (red) and 200 nm (light blue).

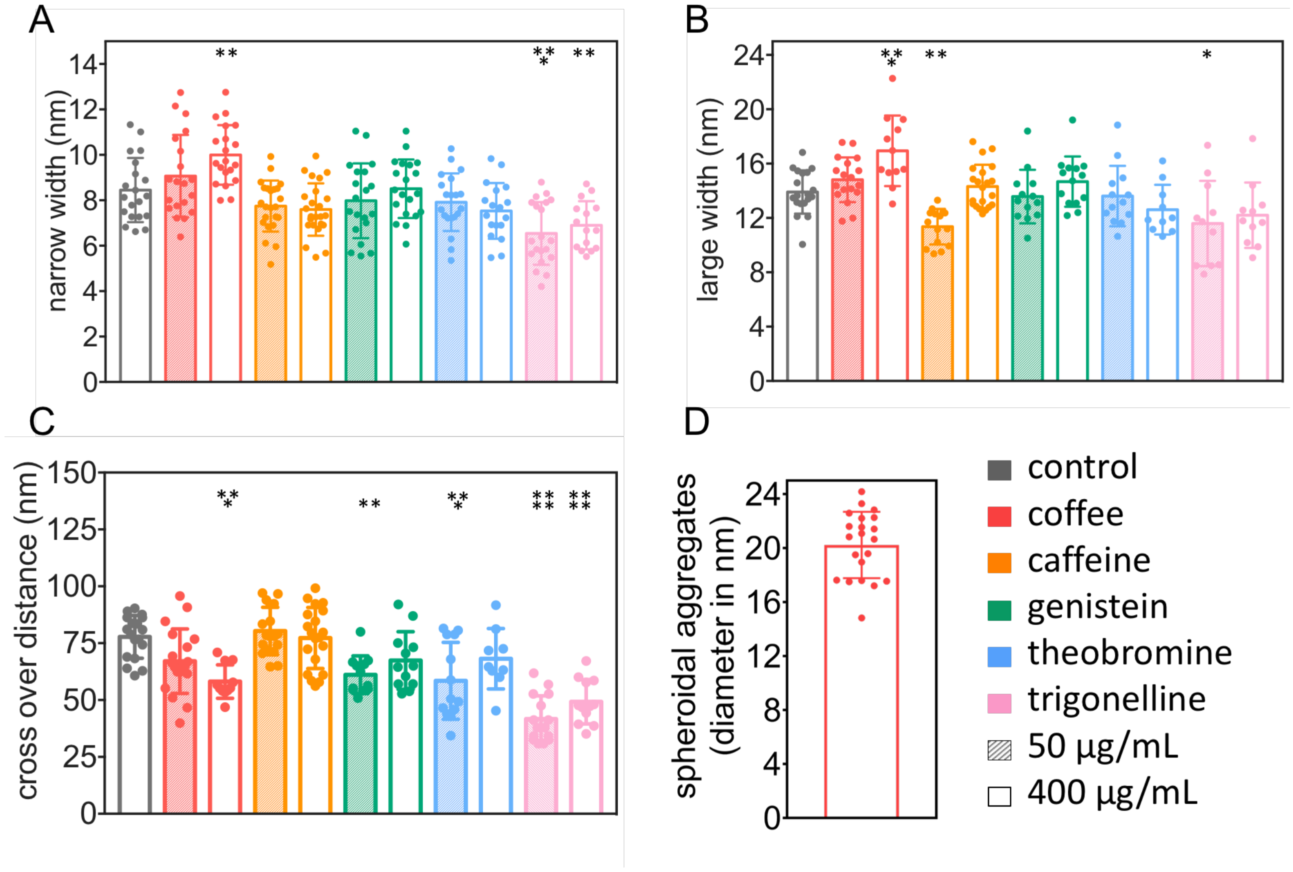


**Figure S7.** *Quantitative analysis of aggregates morphology*. Distributions of (A) narrow width, (B) large width, (C) crossover distance of aggregates obtained in the absence or in the presence of different bioactive molecules. (D) Diameter of the spheroidal aggregates formed in the presence of high coffee-extract concentration. Data are presented as column scatter charts, mean ± s.d. of 10-20 values for each parameter are displayed. Distributions were compared to control data by one-way ANOVA followed by Dunnett's multiple comparison test, P = * 0.01-0.05, ** 0.001-0.01, *** 0.0001-0.001, and **** < 0.0001.

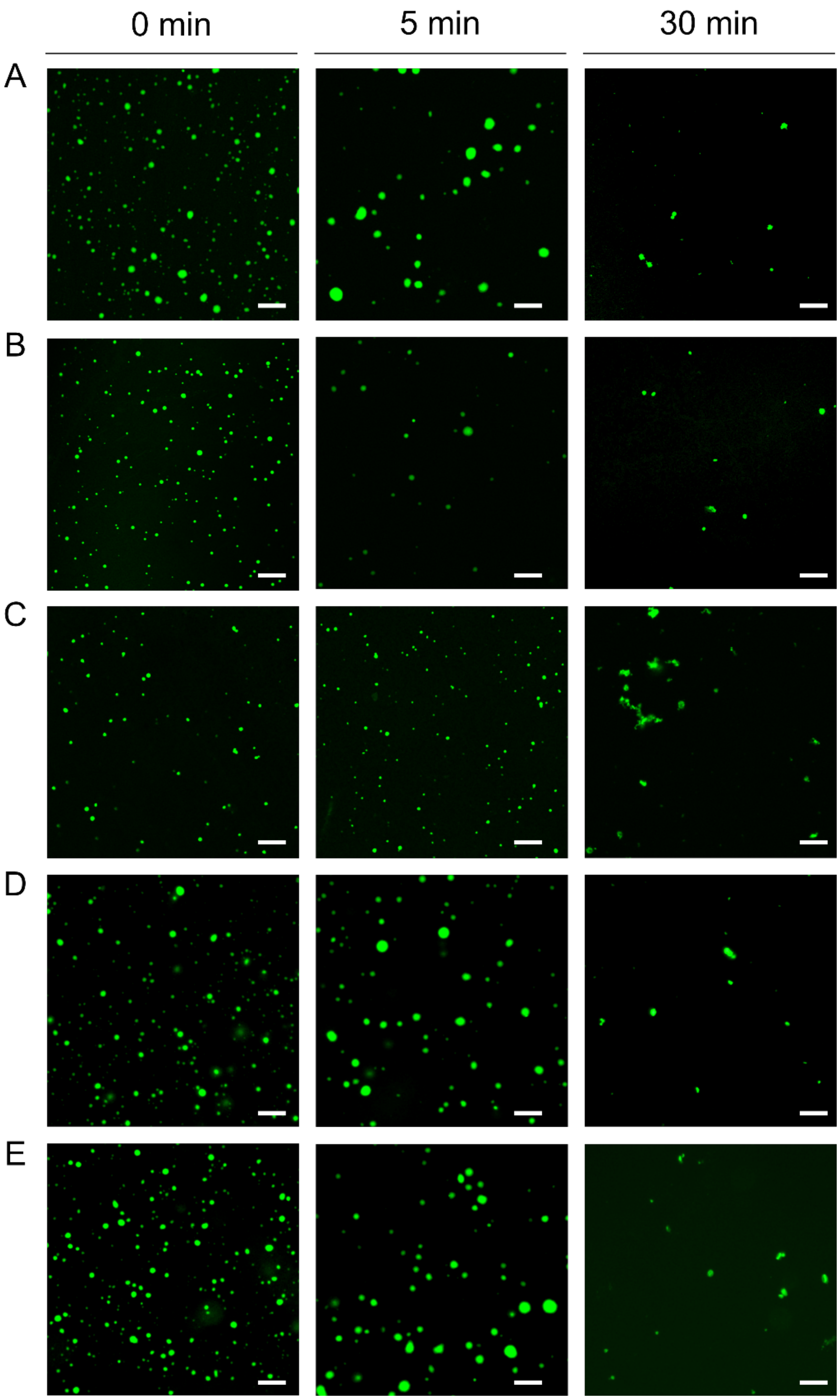


**Figure S8**. *Influence of coffee extract on tau condensates*. Representative fluorescence microscopy images displaying condensates of tau$^{4RD}$/heparin in simple buffer (A), in the presence of 35 µg/mL coffee extract (B), 280 µg/mL coffee extract (C), 35 µg/mL coffee extract added 5 min after mixing tau and heparin (D), 280 µg/mL coffee extract added 5 min after mixing tau and heparin (E). Images were acquired at 0, 5, 30 min after sample preparation. Protein was 35 µM and heparin 8.75 µM. Scale bar is 10 µm.

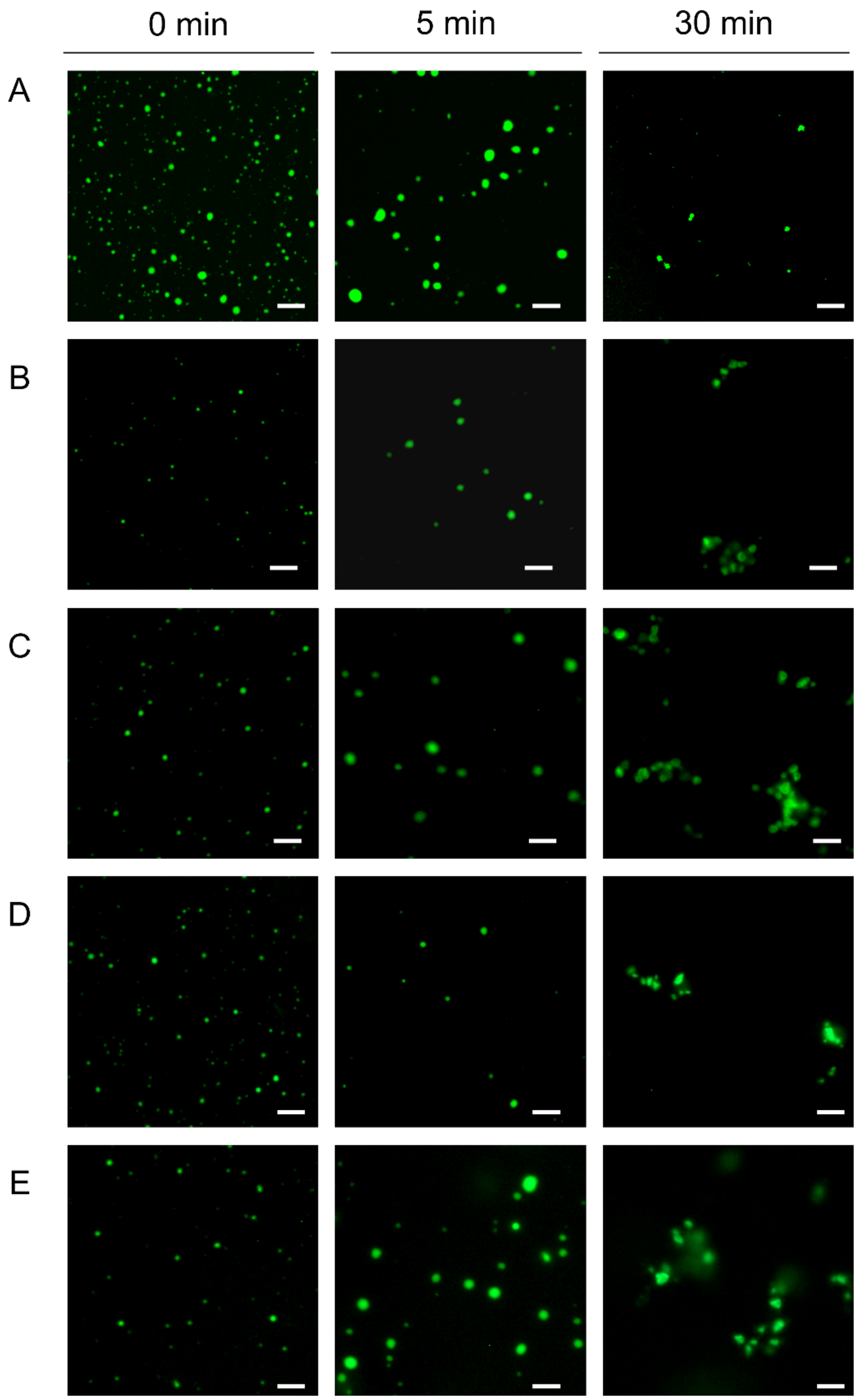


**Figure S9.** *Influence of caffeine on tau condensates*. Representative fluorescence microscopy images displaying condensates of tau$^{4RD}$/heparin in simple buffer (A), in the presence of 35 µg/mL (0.18 mM) caffeine (B), 280 µg/mL (1.4 mM) caffeine (C), 35 µg/mL caffeine added 5 min after mixing tau and heparin (D), 280 µg/mL caffeine added 5 min after mixing tau and heparin (E). Images were acquired at 0, 5, 30 min after sample preparation. Protein was 35 µM and heparin 8.75 µM. Scale bar is 10 µm.

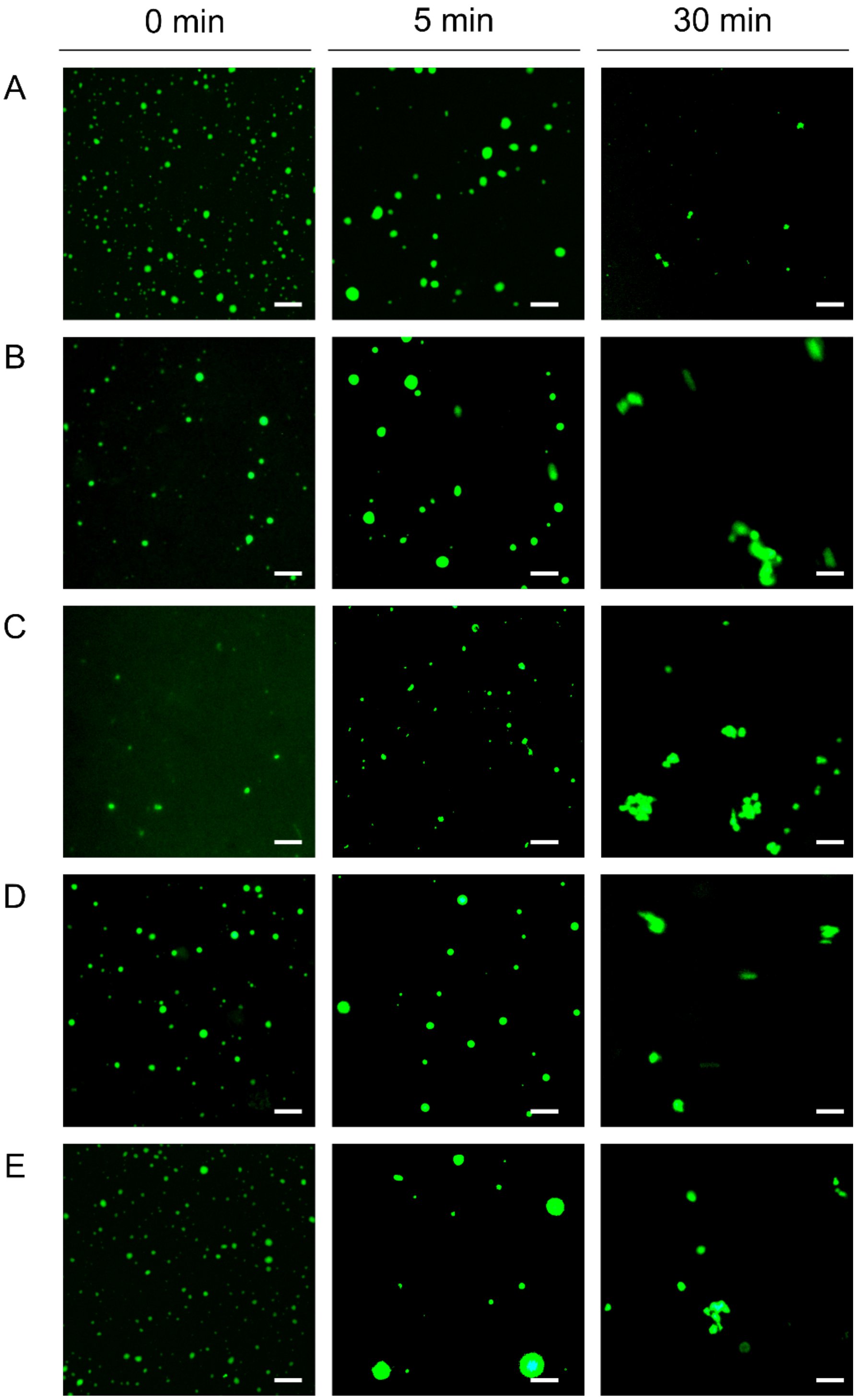


**Figure S10**. *Influence of genistein on tau condensates*. Representative fluorescence microscopy images displaying condensates of tau$^{4RD}$/heparin in simple buffer (A), in the presence of 35 µg/mL (0.13 mM) genistein (B), 280 µg/mL (1 mM) genistein (C), 35 µg/mL genistein added 5 min after mixing tau and heparin (D), 280 µg/mL genistein added 5 min after mixing tau and heparin (E). Images were acquired at 0, 5, 30 min after sample preparation. Protein was 35 µM and heparin 8.75 µM. Scale bar is 10 µm.

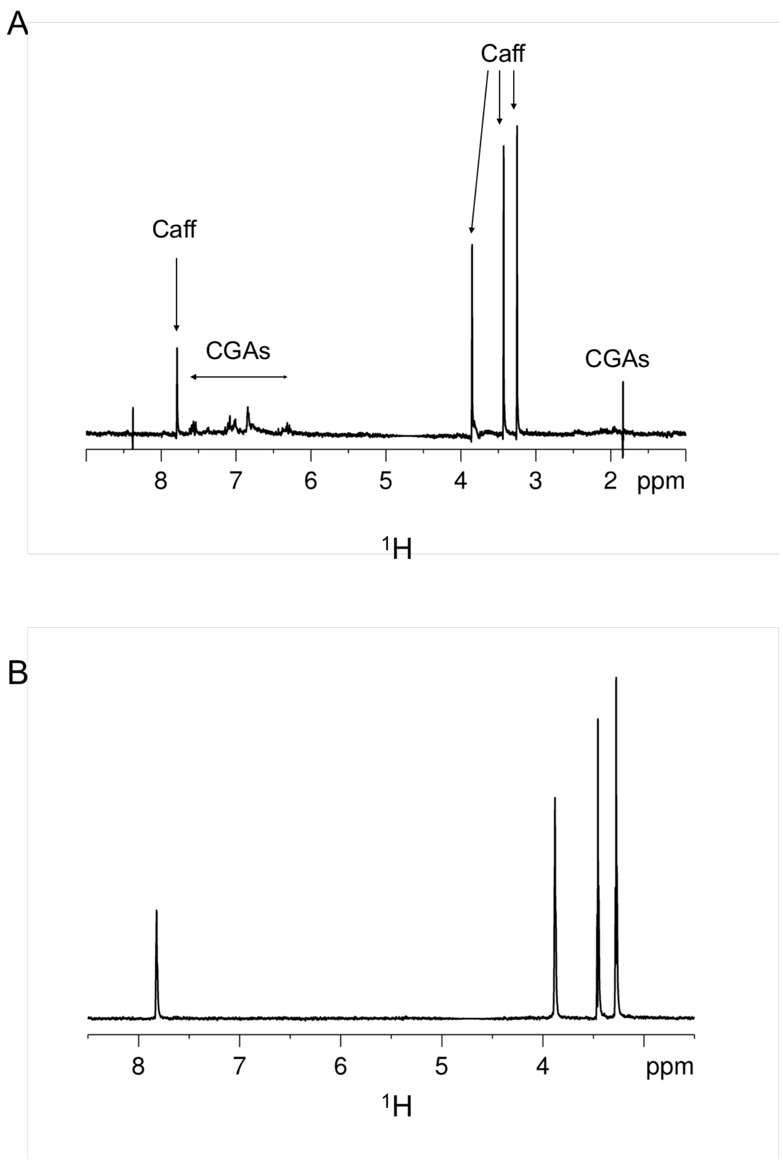


**Figure S11.** *Interaction of coffee compounds with tau aggregates.* Spectra derived by subtraction of STD spectra of 5 mg/mL coffee (A) or 0.8 mg/mL caffeine (B) in the presence of fibrils from STD spectra recorded in the absence of fibrils.

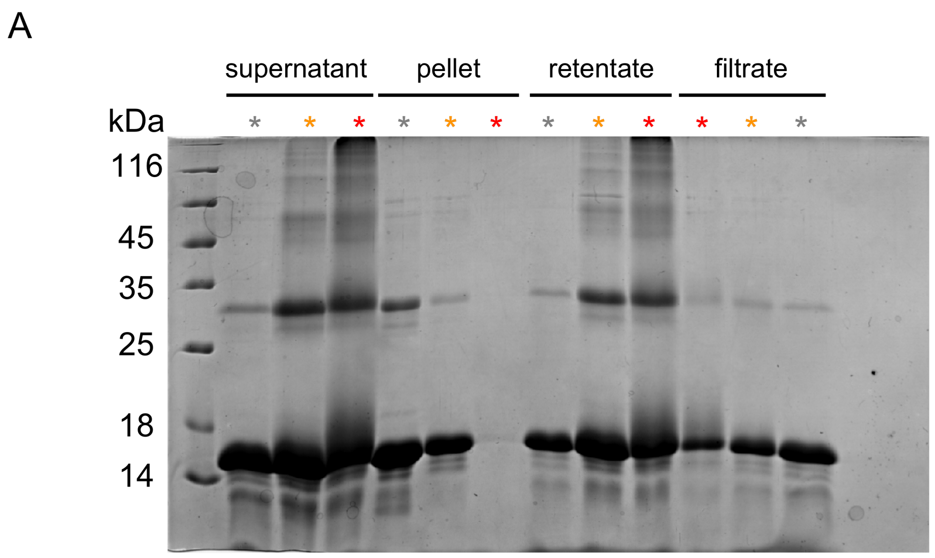


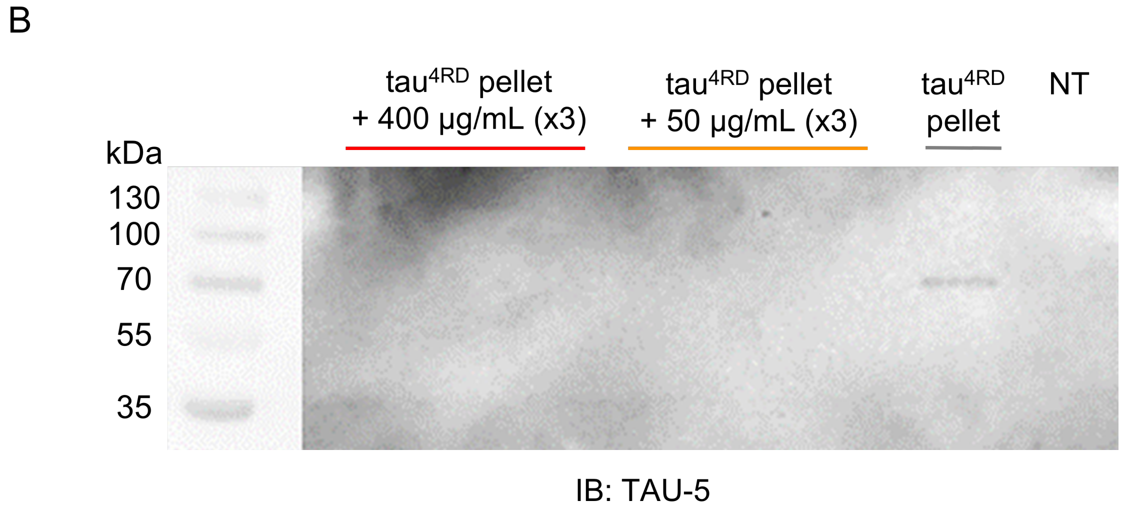


**Figure S12.** *SDS-PAGE and immunoblot analysis of samples of in cell experiments.* (A) SDS-PAGE analysis of samples of tau$^{4RD}$ aggregated in the absence (grey) or in the presence of 50 µg/mL (orange) or 400 µg/mL (red) of coffee extract, at different separation steps as depicted in Fig. 7A. (B) Immunoblot analysis of the cellular insoluble fraction after treatment with the pellets of tau$^{4RD}$ aggregated in buffer and in the presence of 50 µg/mL or 400 µg/mL of coffee extracts. HEK293 cells overexpressing tau$^{FL}$/P310L-GFP, were treated with the samples for 48 h and the Triton-insoluble fractions were blotted with TAU-5 antibody. Last lane represents cells without the treatment (NT).
